# Optical coherence tomography for glaucoma diagnosis: An evidence based meta-analysis

**DOI:** 10.1371/journal.pone.0190621

**Published:** 2018-01-04

**Authors:** Vinay Kansal, James J. Armstrong, Robert Pintwala, Cindy Hutnik

**Affiliations:** 1 University of Saskatchewan, Department of Ophthalmology, Saskatoon, Canada; 2 Western University Canada, Faculty of Medicine, London, Canada; 3 Western University Canada, Department of Ophthalmology, London, Canada; 4 Ivey Eye Institute, St. Joseph’s Hospital, London, Canada; University of Michigan, UNITED STATES

## Abstract

**Purpose:**

Early detection, monitoring and understanding of changes in the retina are central to the diagnosis of glaucomatous optic neuropathy, and vital to reduce visual loss from this progressive condition. The main objective of this investigation was to compare glaucoma diagnostic accuracy of commercially available optical coherence tomography (OCT) devices (Zeiss Stratus, Zeiss Cirrus, Heidelberg Spectralis and Optovue RTVue, and Topcon 3D-OCT).

**Patients:**

16,104 glaucomatous and 11,543 normal eyes reported in 150 studies.

**Methods:**

Between Jan. 2017 and Feb 2017, MEDLINE^®^, EMBASE^®^, CINAHL^®^, Cochrane Library^®^, Web of Science^®^, and BIOSIS^®^ were searched for studies assessing glaucoma diagnostic accuracy of the aforementioned OCT devices. Meta-analysis was performed pooling area under the receiver operating characteristic curve (AUROC) estimates for all devices, stratified by OCT type (RNFL, macula), and area imaged.

**Results:**

150 studies with 16,104 glaucomatous and 11,543 normal control eyes were included. Key findings: AUROC of glaucoma diagnosis for RNFL average for all glaucoma patients was 0.897 (0.887–0.906, n = 16,782 patient eyes), for macula ganglion cell complex (GCC) was 0.885 (0.869–0.901, n = 4841 eyes), for macula ganglion cell inner plexiform layer (GCIPL) was 0.858 (0.835–0.880, n = 4211 eyes), and for total macular thickness was 0.795 (0.754–0.834, n = 1063 eyes).

**Conclusion:**

The classification capability was similar across all 5 OCT devices. More diagnostically favorable AUROCs were demonstrated in patients with increased glaucoma severity. Diagnostic accuracy of RNFL and segmented macular regions (GCIPL, GCC) scans were similar and higher than total macular thickness. This study provides a synthesis of contemporary evidence with features of robust inclusion criteria and large sample size. These findings may provide guidance to clinicians when navigating this rapidly evolving diagnostic area characterized by numerous options.

## Introduction

Glaucoma is the leading cause of irreversible blindness worldwide[1]. As the population continues to age, and average life expectancies increase, the prevalence of this debilitating disease will grow. Glaucoma is one of the leading causes of blindness in working-age populations of industrialized nations, and is the most common cause of permanent vision loss in persons older than 40 years of age, after age-related macular degeneration[2–4].

Glaucoma is a multifactorial, chronic optic nerve neuropathy that is characterized by progressive loss of retinal ganglion cells (RGC), which leads to structural damage to the optic nerve head (ONH), retinal nerve fiber layer (RNFL), and consequent visual field defects[5]. Early diagnosis and treatment of glaucoma has been shown to reduce the rate of disease progression, and improve patients’ quality of life[6]. The currently accepted gold standards for glaucoma diagnosis are optic disc assessment for structural changes, and achromatic white-on-white perimetry to monitor changes in function[7]. However, imaging technologies such as optic coherence technology (OCT) are playing an increasing role in glaucoma diagnosis, monitoring of disease progress, and quantification of structural damage[8,9].

OCT is a non-invasive, non-contact imaging modality that provides high-resolution cross-sectional imaging of ocular tissues (retina, optic nerve, and anterior segment). Image acquisition is analogous to ultrasound, where light waves is used in lieu of sound waves. Low coherence infrared light is directed toward the tissue being imaged, from which it scatters at large angles. An interferometer (beam splitter) is used to record the path of scattered photons and create three-dimensional images[10–13]. OCT is highly reproducible, and is thus widely used as an adjunct in routine glaucoma patient management[14–16].

Peripapillary RNFL analysis is the most commonly used scanning protocol for glaucoma diagnosis[14–16], as it samples RGCs from the entire retina; however, it does suffer certain drawbacks related to inter-patient variability in ONH morphology[17,18]. To overcome some of these disadvantages, the macular thickness has been proposed as a means of glaucoma detection[19]– 50% of RGCs are found in the macula, and RGC bodies are thicker than their axons, thus are potentially easier to detect. The older time-domain (TD) OCT devices, such as Zeiss Stratus, were able to only measure total macular thickness, which had been shown to have poorer glaucoma diagnostic accuracy than RNFL thickness[20–22]. Spectral-domain (SD) OCT (Zeiss Cirrus, Heidelberg Spectralis, Optovue RTVue, Topcon 3D-OCT) allows for measurement of specific retinal layers implicated in the pathogenesis of glaucoma, namely: macular nerve fiber layer (mNFL), ganglion cell layer with inner plexiform layer (GCIPL), and ganglion cell complex (GCC) (composed of mNFL and GCIPL). Segmented analysis is purported to have better diagnostic ability for glaucoma than total retinal thickness[23,24], and may be comparable to RNFL thickness[23,25,26].

Currently, several OCT devices are available on the market, each with unique technologies purported to provide better clinical information to the user. The technical features of these various systems have been described elsewhere[27,28]. Reichel et al. also provide images obtained from each of the OCT systems[27]. It is unclear however; which OCT device should be selected by practitioners when making referral or treatment decisions. The aim of this meta-analysis was to provide pooled estimates for the accuracy and detection capability of the most commonly used OCT imaging devices (Zeiss Cirrus OCT, Zeiss, Stratus OCT, Heidelberg Spectralis, Optovue RTVue, Topcon 3D-OCT) for glaucoma diagnosis and classification between patients and healthy individuals.

## Methods

### Overview of review methods

**Fig 1:**
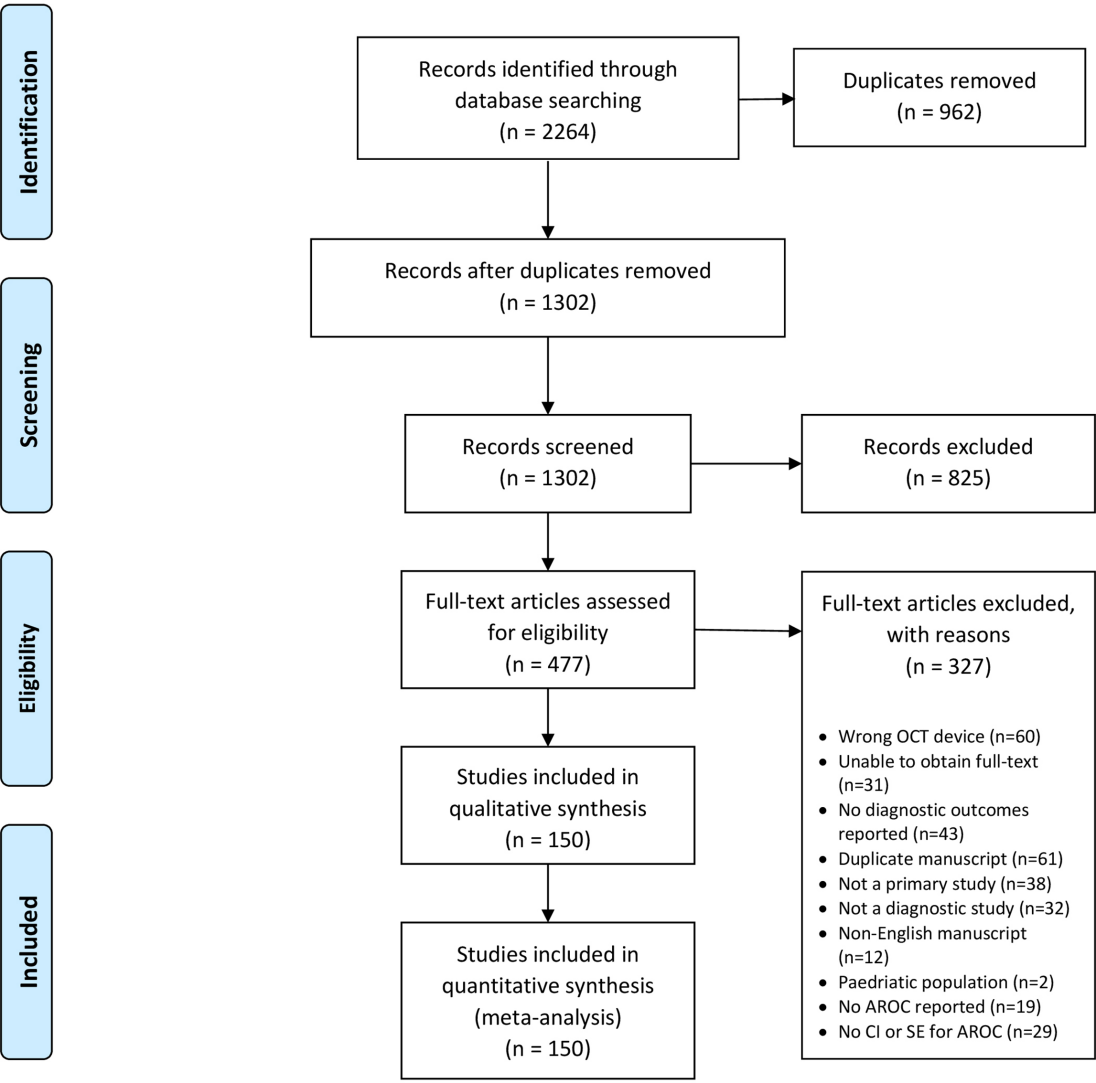
Study flow in this meta-analysis (PRISMA guidelines).

The main objective of this investigation was to compare the glaucoma diagnostic accuracy for each of the OCT devices commercially available, namely Zeiss Stratus, Zeiss Cirrus, Heidelberg Spectralis, Optovue RTVue and Topcon 3D-OCT. We compared diagnostic accuracies of RNFL and macular parameters obtained by these imaging devices. This review was performed in accordance with the Preferred Reporting Items for Systematic Reviews and Meta-Analyses (PRISMA) statement methodology[29]. A PRISMA flow diagram is used to illustrate the flow of records throughout this review (Fig 1).

### Data sources and search strategy

The search strategy for this investigation was comprehensive, aiming to retrieve the largest possible number of relevant studies. An electronic search strategy was developed through consultation with an experienced ophthalmologist specializing in glaucoma management. The search end date was February 2017. There was no specified search start date. Any study providing information on area under receiver operating characteristic curve, sensitivity, specificity, negative predictive value, positive predictive value, likelihood ratio, or diagnostic odds ratio was included. Published and unpublished studies were considered.

The following bibliographic databases were searched: MEDLINE^®^ (Ovid MEDLINE(R) Epub Ahead of Print, In-Process & Other Non-Indexed Citations, Ovid MEDLINE(R) Daily, Ovid MEDLINE and Versions(R)), EMBASE^®^ (Embase Classic+Embase), CINAHL^®^, Cochrane Library^®^ (Wiley Library), Web of Science^®^, and BIOSIS^®^. Specific keywords used in the search included terms for glaucoma, optical coherence tomography, imaging device manufacturer (ie. Zeiss, Heidelberg, RTVue, Topcon), and diagnostic testing including terms for diagnostic evaluative tests (ie. Area under receiver operating characteristic curve, etc.). Search strategies for each of the devices are available in S1 Table (Appendix 1).

### Inclusion and exclusion criteria

All studies that assessed the diagnostic accuracy of OCT for detection of glaucoma were considered for inclusion in our review. As the goal of this investigation was to maximize generalizability and applicability to clinical practice, a broad gold standard was accepted for inclusion, ie. White on white automated perimetry, optic disc appearance (clinically or by photograph), or combination thereof. Accepting a wider gold standard more accurately reflects the reality of clinical practice, and allowed for inclusion of a larger number of articles, improving robustness of the quantitative meta-analysis. Only human, clinical studies published in English-language were accepted. Patient were 18 years of age or greater. No exclusions were made for patient ethnicity, or country where study was conducted. Included studies assessed at least one of five devices, namely Stratus OCT (Carl Zeiss Meditec, Jena, Germany), Cirrus OCT (Carl Zeiss Meditec), Spectralis OCT (Heidelberg Engineering Inc., Heidelberg, Deutschland), RTVue (Optovue Inc., Freemont, United States), and 3D-OCT (Topcon, Tokyo, Japan). These devices were included as they represent the newest or most widely utilized OCT devices available for glaucoma diagnosis at the time of this review. Studies of both RNFL and macular areas for glaucoma diagnosis were included.

During full-text screening, articles were included if they reported area under receiver operating characteristic curve (AUROC) statistics. Manuscripts that did not report standard error or confidence intervals for AUROC were excluded. Other exclusions were: duplicate manuscripts, non-diagnostic studies, studies of pediatric patients, studies without control participants, and investigations of OCT devices other than those previously specified.

### Study selection

All studies included for consideration underwent two levels of screening by two independent reviewers. All records were uploaded to an online interface (Covidence, Veritas Health Innovation, Melbourne, Australia) to coordinate and support the screening process. First, a broad screen of titles, keywords and abstracts (Level 1) was performed. At this stage, studies were tagged as either “Relevant”, “Irrelevant” or “Maybe Relevant”. For all relevant studies, full text screening was performed (Level 2) using the stricter *a priori* inclusion criteria detailed previously.

After each level of screening, disagreements between article screeners were resolved through consultation with the primary author. Reasons for exclusion were documented and are reported in the review. The PRISMA flow chart of studies during screening is illustrated in Fig 1.

### Data extraction and quality assessment

An electronic data extraction form specific to this meta-analysis was developed *a priori*. Data collected included study identification information (title, authors, journal and year of publication, study methodology (design, inclusion/exclusion criteria, gold standard type), patient variables (number of patients/controls, glaucoma diagnosis, age, gender), OCT device used, area imaged (RNFL, macula subtype), and AUROC (with SE/CI).

**Fig 2:**
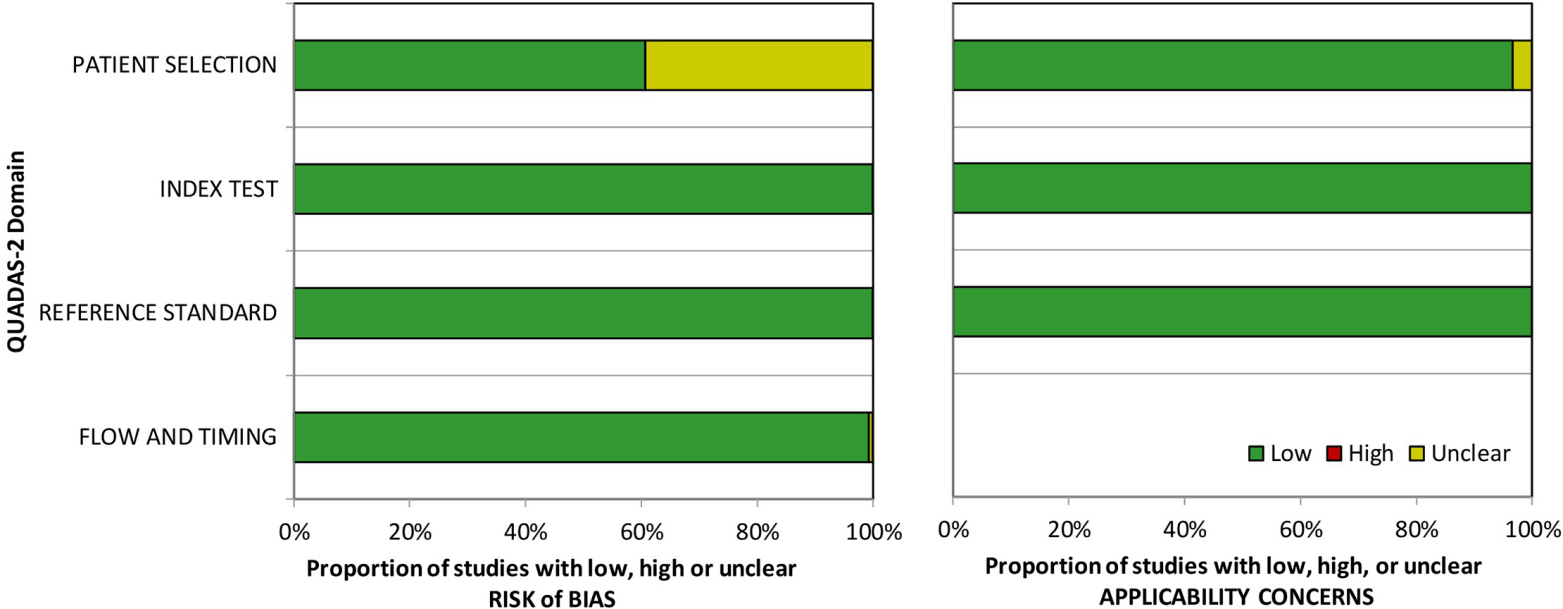
Methodological quality of included studies using the QUADAS 2 tool.

The quality assessment of diagnostic accuracy studies, version 2 (QUADAS-2)[30] was used to assess the risk of bias and applicability concerns of all manuscripts included in this review. This assessment tool comprises four key domains: 1) patient selection, 2) index test, 3) reference standard, and 4) flow of patients through the study and timing between index test and reference standard. Each domain was assessed in terms of risk of bias. The first three domains were assessed for their applicability to the research question being assessed by the review. Results of QUADAS-2 are summarized in Fig 2.

### Data synthesis and statistical analysis

All statistical analyses were performed using MedCalc (Version 17.2, MedCalc Software, Ostend, Belgium). Meta-analysis for the AUROC was selected instead of other measures such as sensitivity and specificity. The AUROC is a commonly used metric for diagnostic accuracy of medical tests. It was found to be more consistently reported in the included studies. Whereas some studies reported a combination of parameters, others reported sensitivity values for particular specificity cut-offs, which, in turn, were not consistent across studies. AUROC reflects both the sensitivity and specificity of a diagnostic test, can be compared across studies, and can be combined between similar studies when measures of uncertainty (standard error (SE) or confidence interval (CI)) are provided[31].

Meta-analysis was completed using MedCalc (MedCalc, Version 17.2, MedCalc Software, Ostend, Belgium). The main outcome of this study was pooled AUROC for each of the following groups: all glaucoma patients, perimetric glaucoma, pre-perimetric, mild glaucoma, moderate to severe glaucoma, and myopic glaucoma. As there currently does not exist any international consensus on the definition of glaucoma severity, there was heterogeneity in the way that each study defined their patient groups. For consistency, we defined each group as follows: 1) *Perimetric glaucoma–*glaucoma based on abnormal visual field measurements; 2) *Pre-perimetric glaucoma*–glaucoma diagnosed based on optic disc appearance, with normal visual field measurements; 3) *Mild glaucoma*–perimetric glaucoma, defined as mean deviation of > -6.00 dB as per the Hodapp-Parrish-Anderson criteria[32]. Patients with normal visual fields were not included in this group; 4) *Moderate to severe glaucoma*–perimetric glaucoma, defined as mean deviation < -6.00 dB[32]; 5) *Myopic glaucoma*–any definition of myopia as defined by study authors, this could include dioptric definition (ex. Spherical equivalent < -6.0) or axial length definition (AL >25mm).

Individual measures of AUROC from each study were pooled into a weighted summary AUROC for each group using the methods described in Zhou et al.[31] Heterogeneity among included studies was tested by computing the I^2^, Z-value and χ2 statistics. An I^2^ value of less than 50% implies low heterogeneity and supports the use of a fixed-effect meta-analysis model. A value of greater than or equal to 50% implies high heterogeneity and supports the use of a random-effects model. Additionally, a high Z-value, a low p-value (<0.01) and a large χ2 value implies significant heterogeneity and supports the use of a random-effects model using DerSimonian and Laird methods. Forest plots were generated to visualize results. Publication bias was assessed through evaluation of funnels plots of included studies for each pooled AUROC.

## Results

### Search results and study characteristics

Study flow is summarized in Fig 1. After removal of duplicates, 1301 records underwent title and abstract (Level 1) screening. 825 were excluded as irrelevant. The remaining 477 records underwent full-text screening (Level 2). Of these, 327 articles were excluded as they did not meet the study inclusion criteria, or manuscript was unable to be obtained. At the end of screening, 150 articles were included for meta-analysis [21–24,33–178].

Characteristics of the 150 included studies are presented in S2 Table (Appendix 2). 67 (44.7%) of studies were case-control studies, 73 (48.7%) were cross-sectional studies, and 10 (6.7%) were cohort studies. 34 (22.7%) used visual field as a reference standard, 6 (4.0%) used disc appearance, 110 (73.3%) used a combination of structural and functional criteria. 55 studies examined the Zeiss Cirrus OCT, 49 studies assessed Zeiss Stratus OCT, 23 studies evaluated Heidelberg Spectralis, 38 studies examined Optovue RTVue, and 14 studies evaluated the Topcon 3D-OCT. There were 50.0% male, and 50.0% female glaucoma patients (reported in 150 studies). Controls were 46.5% male, 53.5% female (reported in 109 studies). The mean age of glaucoma patients was 58.8 ± 11.2, of controls was 54.1 ± 11.1 (Table 1).

**Table 1:** Summarized study and patient characteristics.

				Gender	
	# of eyes	# of Studies	Age ± SD (# of study groups, # of studies)	Male (%) (# of study groups, # of studies)	Female (%) (# of study groups, # of studies)
**Patient groups**					
Normal Controls	11543	150	54.1 ± 11.1 (141,141)	3683 (46.5%) (109,109)	4232 (53.5%) (109,109)
All Glaucoma Patients	16103	150	58.8 ± 11.2 (214,137)	5255 (49.3%) (158,103)	5403 (50.7%) (158,103)
Perimetric (severity unspecified)	10335	122	60.1 ± 11.3 (108,96)	3196 (49.6%) (77,70)	3248 (50.4%) (77,70)
Preperimetric	1711	39	56.4 ± 10.7 (32,29)	502 (42.7%) (23,22)	673 (57.3%) (23,22)
Mild	2369	40	57 ± 11.3 (35,30)	829 (50.2%) (28,23)	823 (49.8%) (28,23)
Moderate to Severe	1199	24	60.4 ± 11.4 (18,10)	325 (51.2%) (15,8)	310 (48.8%) (15,8)
Myopic	358	9	45.3 ± 10.6 (8,7)	194 (58.8%) (7,7)	136 (41.2%) (7,7)
**OCT Device**					
Cirrus	7362	53	57.4 ± 11.9 (75,49)	2249 (49.7%) (50,36)	2273 (50.3%) (50,36)
Stratus	3120	42	58.9 ± 10.2 (47,37)	1083 (48.7%) (37,28)	1141 (51.3%) (37,28)
Spectralis	1710	20	62.7 ± 10.5 (25,20)	668 (52.4%) (20,16)	606 (47.6%) (20,16)
RTVue	3048	30	59.5 ± 11.1 (47,26)	993 (47%) (41,20)	1119 (53%) (41,20)
3D-Topcon	863	10	59.7 ± 11.5 (15,10)	262 (49.8%) (9,6)	264 (50.2%) (9,6)
**Imaged Regions**					
RNFL	13089	130	58.7 ± 11.2 (162,117)	4213 (49.8%) (117,87)	4245 (50.2%) (117,87)
Macula–GCIPL	1217	6	59.9 ± 12.9 (13,5)	331 (52.9%) (8,4)	295 (47.1%) (8,4)
Macula–GCC	1075	9	59.7 ± 10.9 (17,8)	392 (42.3%) (17,8)	535 (57.7%) (17,8)
Macula—mNFL	237	3	58.6 ± 11.8 (5,3)	84 (42.2%) (4,2)	115 (57.8%) (4,2)
Macula–Total thickness	485	7	58.1 ± 8.9 (12,7)	235 (52.5%) (11,5)	213 (47.5%) (11,5)

### Study quality

A summary of the methodological quality assessment for included studies is provided in Fig 2. Overall methodological quality of all included studies was strong in terms of risk of bias and applicability to the research question. Of note, there was an unclear risk of bias in patient selection for 39.3% of studies. This was largely due to inadequate reporting of patient selection methods in these manuscripts; thus, risk of bias was unable to be ascertained.

### Diagnostic accuracy of OCT for all glaucoma patients, RNFL and macular parameters

**Fig 3:**
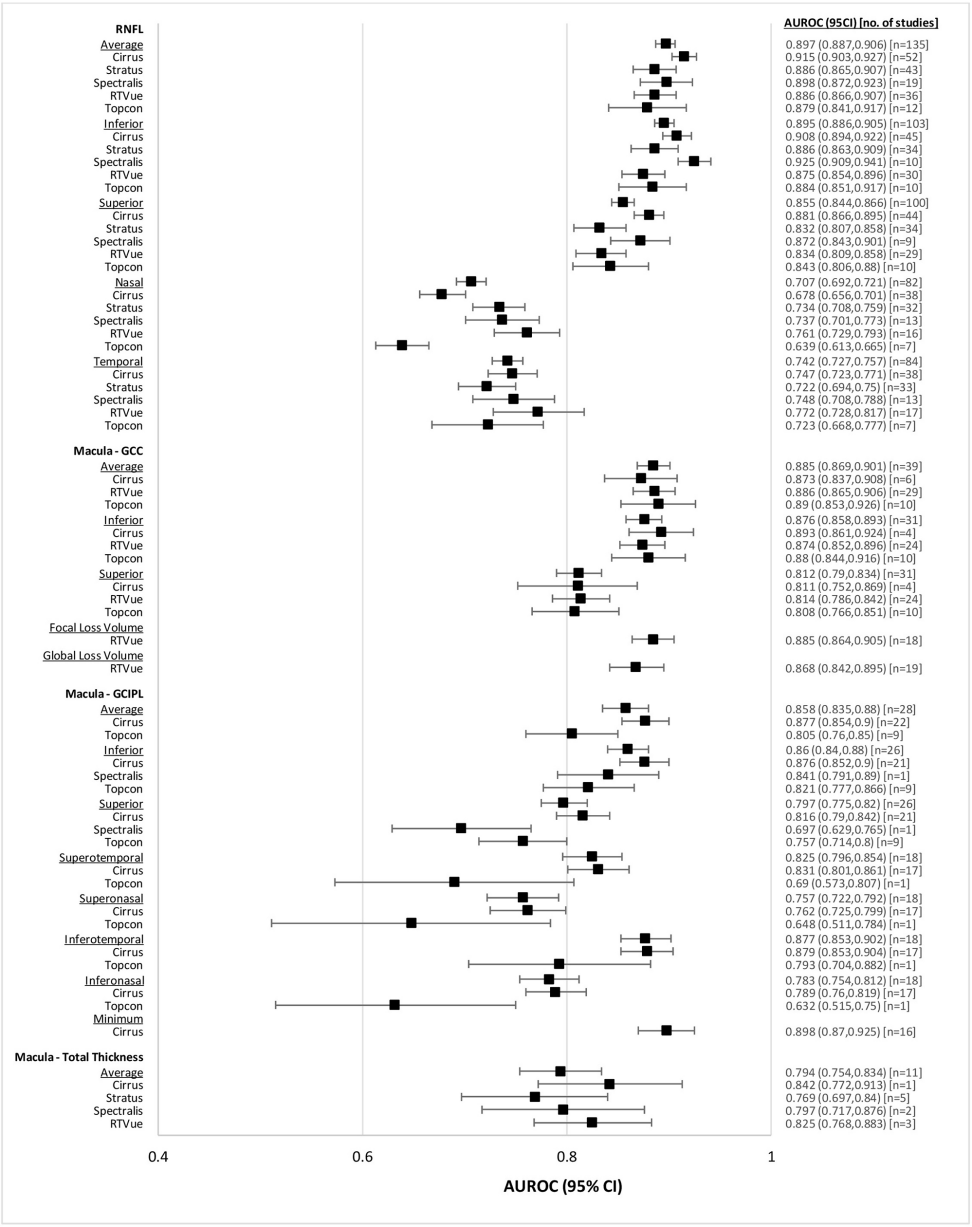
Forest plot of diagnostic accuracies of RNFL and macular OCT parameters, all glaucoma patients.

The diagnostic accuracy of OCT for all glaucoma patients stratified by imaged area and device is reported in Table 2, and displayed graphically in Fig 3. Pooled AUROC ranged from 0.632 to 0.915 depending on imaging device and area imaged. Overall, there were no statistically significance differences between devices for any particular area imaged. Within RNFL parameters, we found that AUROC for glaucoma diagnosis was higher for average (0.897, CI95% 0.887 to 0.906), superior (0.855, CI95% 0.844 to 0.866) and inferior (0.895, CI95% 0.886 to 0.905) areas than nasal (0.707, CI95% 0.692 to 0.721) and temporal (0.742, CI95% 0.727 to 0.757) parameters. For the Macular GCIPL scans, average (0.858, CI95% 0.835 to 0.880), inferior (0.860, CI95% 0.840 to 0.880), temporal (superotemporal (0.825, CI95% 0.796 to 0.854), inferotemporal (0.877, CI95% 0.853 to 0.902)) and minimum parameters had higher AUROC for glaucoma diagnosis than nasal (superonasal (0.757, CI95% 0.722 to 0.792, inferonasal (0.783, CI95% 0.754 to 0.812)) areas. By comparison, there were no major differences between areas for the macular GCC scans.

**Table 2:** Pooled AUROCs of RNFL and macular OCT parameters for all glaucoma patients.

All Glaucoma Patients–Pooled AUROCs (if I^2^ > 50% random effects meta-analysis was used, if I^2^ < 50% fixed effects was used)											
Test Parameter, Location and OCT Device	Number of Studies	Number of Study Groups*	Pooled Sample Size (controls)	Pooled AUROC	95% CI	Test Parameter, Location and OCT Device	Number of Studies	Number of Study Groups*	Pooled Sample Size (eyes)	Pooled AUROC	95% CI
**RNFL**						**Macula—GCIPL**					
Average	*135*	*236*	*16*,*782 (18*,*490)*	*0*.*897*	*0*.*887 to 0*.*906*	Average	28	50	4,211 (4,401)	0.858	0.835 to 0.880
*Cirrus*	*52*	*82*	*6*,*924 (8*,*569)*	*0*.*915*	*0*.*903 to 0*.*927*	*Cirrus*	*22*	*34*	*3062 (3483)*	*0*.*877*	*0*.*854 to 0*.*900*
*Stratus*	*43*	*56*	*3*,*447 (3746)*	*0*.*886*	*0*.*865 to 0*.*907*	*Topcon*	*9*	*15*	*1072 (859)*	*0*.*805*	*0*.*760 to 0*.*850*
*Spectralis*	*19*	*28*	*1682 (1988)*	*0*.*898*	*0*.*872 to 0*.*923*	Inferior	26	54	4,106 (4,428)	0.860	0.840 to 0.880
*RTVue*	*36*	*52*	*3540 (3255)*	*0*.*886*	*0*.*866 to 0*.*907*	*Cirrus*	*21*	*36*	*2950 (3381)*	*0*.*876*	*0*.*852 to 0*.*900*
*Topcon*	*12*	*18*	*1189 (932)*	*0*.*879*	*0*.*841 to 0*.*917*	*Spectralis*	*1*	*2*	*120 (120)*	*0*.*841*	*0*.*791 to 0*.*890*
Inferior	*103*	*183*	*13*,*265 (14*,*580)*	*0*.*895*	*0*.*886 to 0*.*905*	*Topcon*	*9*	*16*	*1036 (927)*	*0*.*821*	*0*.*777 to 0*.*866*
*Cirrus*	*45*	*69*	*5701 (6862)*	*0*.*908*	*0*.*894 to 0*.*922*	Superior	26	53	4,038 (4,364)	0.797	0.775 to 0.820
*Stratus*	*34*	*43*	*2701 (3101)*	*0*.*886*	*0*.*863 to 0*.*909*	*Cirrus*	*21*	*36*	*2950 (3381)*	*0*.*816*	*0*.*790 to 0*.*842*
*Spectralis*	*10*	*16*	*920 (1045)*	*0*.*925*	*0*.*909 to 0*.*941*	*Spectralis*	*1*	*2*	*120 (120)*	*0*.*697*	*0*.*629 to 0*.*765*
*RTVue*	*30*	*39*	*2941 (2707)*	*0*.*875*	*0*.*854 to 0*.*896*	*Topcon*	*9*	*15*	*968 (863)*	*0*.*757*	*0*.*714 to 0*.*800*
*Topcon*	*10*	*16*	*1002 (865)*	*0*.*884*	*0*.*851 to 0*.*917*	Superotemporal	18	30	2,315 (2,336)	0.825	0.796 to 0.854
Superior	*100*	*178*	*12*,*873 (14*,*207)*	*0*.*855*	*0*.*844 to 0*.*866*	*Cirrus*	*17*	*27*	*2*,*064 (2*,*195)*	*0*.*831*	*0*.*801 to 0*.*861*
*Cirrus*	*44*	*66*	*5505 (6698)*	*0*.*881*	*0*.*866 to 0*.*895*	*Topcon*	*1*	*2*	*174 (82)*	*0*.*690*	*0*.*573 to 0*.*807*
*Stratus*	*34*	*43*	*2701 (3101)*	*0*.*832*	*0*.*807 to 0*.*858*	Superonasal	18	30	2,315 (2,336)	0.757	0.722 to 0.792
*Spectralis*	*9*	*15*	*887 (1013)*	*0*.*872*	*0*.*843 to 0*.*901*	*Cirrus*	*17*	*27*	*2*,*064 (2*,*195)*	*0*.*762*	*0*.*725 to 0*.*799*
*RTVue*	*29*	*38*	*2778 (2530)*	*0*.*834*	*0*.*809 to 0*.*858*	*Topcon*	*1*	*2*	*174 (82)*	*0*.*648*	*0*.*511 to 0*.*784*
*Topcon*	*10*	*16*	*1002 (865)*	*0*.*843*	*0*.*806 to 0*.*880*	Inferotemporal	18	30	2,315 (2,336)	0.877	0.853 to 0.902
Nasal	*82*	*147*	*10*,*409 (10*,*838)*	*0*.*707*	*0*.*692 to 0*.*721*	*Cirrus*	*17*	*27*	*2*,*064 (2*,*195)*	*0*.*879*	*0*.*853 to 0*.*904*
*Cirrus*	*38*	*58*	*4719 (4806)*	*0*.*678*	*0*.*656 to 0*.*701*	*Topcon*	*1*	*2*	*174 (82)*	*0*.*793*	*0*.*704 to 0*.*882*
*Stratus*	*32*	*41*	*2501 (2860)*	*0*.*734*	*0*.*708 to 0*.*759*	Inferonasal	18	30	2,315 (2,336)	0.783	0.754 to 0.812
*Spectralis*	*13*	*19*	*1127 (1322)*	*0*.*737*	*0*.*701 to 0*.*773*	*Cirrus*	*17*	*27*	*2*,*064 (2*,*195)*	*0*.*789*	*0*.*760 to 0*.*819*
*RTVue*	*16*	*18*	*1268 (1215)*	*0*.*761*	*0*.*729 to 0*.*793*	*Topcon*	*1*	*2*	*174 (82)*	*0*.*632*	*0*.*515 to 0*.*750*
*Topcon*	*7*	*11*	*794 (635)*	*0*.*639*	*0*.*613 to 0*.*665*	Minimum					
Temporal	*84*	*149*	*10*,*616 (10*,*969)*	*0*.*742*	*0*.*727 to 0*.*757*	*Cirrus*	16	24	1,948 (2,054)	0.898	0.870 to 0.925
*Cirrus*	*38*	*58*	*4719 (4806)*	*0*.*747*	*0*.*723 to 0*.*771*						
*Stratus*	*33*	*42*	*2562 (2917)*	*0*.*722*	*0*.*694 to 0*.*750*	**Macula–Total Thickness**					
*Spectralis*	*13*	*19*	*1127 (1322)*	*0*.*748*	*0*.*708 to 0*.*788*	Average	11	20	1,063 (816)	0.794	0.754 to 0.834
*RTVue*	*17*	*19*	*1414 (1289)*	*0*.*772*	*0*.*728 to 0*.*817*	*Cirrus*	*1*	*2*	*96 (70)*	*0*.*842*	*0*.*772 to 0*.*913*
*Topcon*	*7*	*11*	*794 (635)*	*0*.*723*	*0*.*668 to 0*.*777*	*Stratus*	*5*	*8*	*359 (354)*	*0*.*769*	*0*.*697 to 0*.*840*
						*Spectralis*	*2*	*2*	*140 (73)*	*0*.*797*	*0*.*717 to 0*.*876*
**Macula—GCC**						*RTVue*	*3*	*7*	*438 (284)*	*0*.*825*	*0*.*768 to 0*.*883*
Average	*39*	*70*	*4*,*841 (4*,*103)*	*0*.*885*	*0*.*869 to 0*.*901*						
*Cirrus*	*6*	*9*	*675 (495)*	*0*.*873*	*0*.*837 to 0*.*908*						
*RTVue*	*29*	*45*	*3161 (2799)*	*0*.*886*	*0*.*865 to 0*.*906*						
*Topcon*	*10*	*15*	*928 (750)*	*0*.*890*	*0*.*853 to 0*.*926*						
Inferior	*31*	*52*	*3*,*689 (3*,*155)*	*0*.*876*	*0*.*858 to 0*.*893*						
*Cirrus*	*4*	*6*	*530 (363)*	*0*.*893*	*0*.*861 to 0*.*924*						
*RTVue*	*24*	*31*	*2231 (2042)*	*0*.*874*	*0*.*852 to 0*.*896*						
*Topcon*	*10*	*15*	*928 (750)*	*0*.*880*	*0*.*844 to 0*.*916*						
Superior	*31*	*52*	*3689 (3155)*	*0*.*812*	*0*.*790 to 0*.*834*						
*Cirrus*	*4*	*6*	*530 (363)*	*0*.*811*	*0*.*752 to 0*.*869*						
*RTVue*	*24*	*31*	*2231 (2042)*	*0*.*814*	*0*.*786 to 0*.*842*						
*Topcon*	*10*	*15*	*928 (750)*	*0*.*808*	*0*.*766 to 0*.*851*						
Focal Loss Volume											
*RTVue*	*18*	*28*	*1745 (1797)*	*0*.*885*	*0*.*864 to 0*.*905*						
Global Loss Volume											
*RTVue*	*19*	*28*	*2296 (2194)*	*0*.*868*	*0*.*842 to 0*.*895*						

*Certain studies reported outcomes of several glaucoma subgroups.

Comparing the diagnostic efficacy between RNFL and macular thickness, we note that average RNFL (0.897, CI95% 0.887 to 0.906), average macula GCC (0.885, CI95% 0.869 to 0.901), and average macula GCIPL (0.858, CI95% 0.835 to 0.880) thicknesses have similar AUROC for glaucoma diagnosis. By comparison, AUROC of average macular total thickness (0.794, CI95% 0.754 to 0.834) is lower.

### Diagnostic accuracy of OCT for patient subgroups, RNFL and macular parameters

#### Perimetric glaucoma

**Fig 4:**
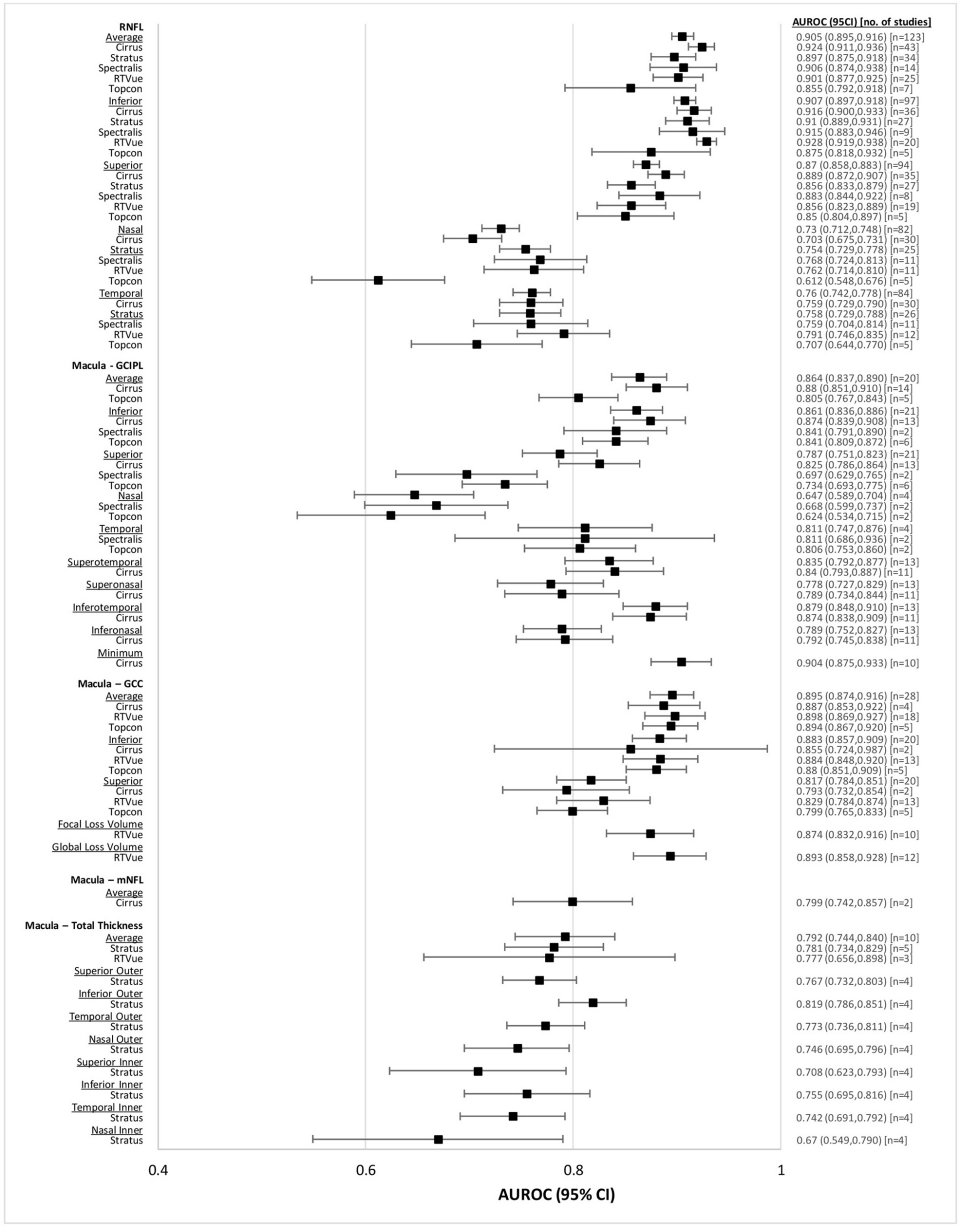
Forest plot of diagnostic accuracies of RNFL and macular OCT parameters, perimetric glaucoma.

Diagnostic accuracy of OCT for patients with perimetric glaucoma is reported in Table 3, and represented in a forest plot in Fig 4. Findings largely mirror what was found for the overall glaucoma population, with AUROCs being higher. All devices performed relatively similarly for glaucoma diagnosis, with the Zeiss Cirrus OCT demonstrating highest AUROC for most RNFL and Macula GCIPL parameters. For the RNFL, average (0.905, CI95% 0.895 to 0.916), superior (0.870, CI95% 0.858 to 0.883), and inferior (0.907, CI95% 0.897 to 0.918) thicknesses had higher AUROC than nasal (0.730, CI95% 0.712 to 0.748) and temporal (0.760, CI95% 0.742 to 0.778) regions. Within macula GCIPL, the Macular GCIPL scans, average (0.864, CI95% 0.837 to 0.890), inferior (0.861, CI95% 0.836 to 0.886), temporal (superotemporal (0.835, CI95% 0.792 to 0.877), inferotemporal (0.879, CI95% 0.848 to 0.910)) and minimum (0.904, CI95% 0.875 to 0.933) parameters had higher AUROC for glaucoma diagnosis than nasal (superonasal (0.778, CI95% 0.727 to 0.829), inferonasal (0.789, CI95% 0.752 to 0.827)) areas. There were no notable differences in AUROC between different macular GCC areas.

**Table 3:** Pooled AUROCs of RNFL and macular OCT parameters for perimetric glaucoma patients.

Perimetric Glaucoma–Pooled AUROCs (if I^2^ > 50% random effects meta-analysis was used, if I^2^ < 50% fixed effects was used)									
Test Parameter, Location and OCT Device	Number of Studies	Pooled Sample Size	Pooled AUROC	95% CI	Test Parameter, Location and OCT Device	Number of Studies	Pooled Sample Size	Pooled AUROC	95% CI
**RNFL**					**Macula–GCC**				
Average	123	10612 (9938)	0.905	0.895 to 0.916	Average	28	2599 (1799)	0.895	0.874 to 0.916
*Cirrus*	*43*	*4310 (4472)*	*0*.*924*	*0*.*911 to 0*.*936*	*Cirrus*	*4*	*347 (209)*	*0*.*887*	*0*.*853 to 0*.*922*
*Stratus*	*34*	*2498 (2416)*	*0*.*897*	*0*.*875 to 0*.*918*	*RTVue*	*18*	*1742 (1237)*	*0*.*898*	*0*.*869 to 0*.*927*
*Spectralis*	*14*	*1023 (944)*	*0*.*906*	*0*.*874 to 0*.*938*	*Topcon*	*5*	*433 (294)*	*0*.*894*	*0*.*867 to 0*.*920*
*RTVue*	*25*	*2161 (1745)*	*0*.*901*	*0*.*877 to 0*.*925*	Inferior	20	1867 (1280)	0.883	0.857 to 0.909
*Topcon*	*7*	*620 (361)*	*0*.*855*	*0*.*792 to 0*.*918*	*Cirrus*	*2*	*251 (128)*	*0*.*855*	*0*.*724 to 0*.*987*
Inferior	97	8352 (7892)	0.907	0.897 to 0.918	*RTVue*	*13*	*1183 (858)*	*0*.*884*	*0*.*848 to 0*.*920*
*Cirrus*	*36*	*3461 (3458)*	*0*.*916*	*0*.*900 to 0*.*933*	*Topcon*	*5*	*433 (294)*	*0*.*880*	*0*.*851 to 0*.*909*
*Stratus*	*27*	*1932 (2027)*	*0*.*910*	*0*.*889 to 0*.*931*	Superior	20	1867 (1280)	0.817	0.784 to 0.851
*Spectralis*	*9*	*645 (603)*	*0*.*915*	*0*.*883 to 0*.*946*	*Cirrus*	*2*	*251 (128)*	*0*.*793*	*0*.*732 to 0*.*854*
*RTVue*	*20*	*1881 (1510)*	*0*.*928*	*0*.*919 to 0*.*938*	*RTVue*	*13*	*1183 (858)*	*0*.*829*	*0*.*784 to 0*.*874*
*Topcon*	*5*	*433 (294)*	*0*.*875*	*0*.*818 to 0*.*932*	*Topcon*	*5*	*433 (294)*	*0*.*799*	*0*.*765 to 0*.*833*
Superior	94	8108 (7648)	0.870	0.858 to 0.883	Focal Loss Volume				
*Cirrus*	*35*	*3413 (3423)*	*0*.*889*	*0*.*872 to 0*.*907*	RTVue	10	836 (663)	0.874	0.832 to 0.916
*Stratus*	*27*	*1932 (2027)*	*0*.*856*	*0*.*833 to 0*.*879*	Global Loss Volume				
*Spectralis*	*8*	*612 (571)*	*0*.*883*	*0*.*844 to 0*.*922*	RTVue	12	1145 (914)	0.893	0.858 to 0.928
*RTVue*	*19*	*1718 (1333)*	*0*.*856*	*0*.*823 to 0*.*889*	**Macula–mNFL**				
*Topcon*	*5*	*433 (294)*	*0*.*850*	*0*.*804 to 0*.*897*	Average				
Nasal	82	6722 (6255)	0.730	0.712 to 0.748	Cirrus	2	140 (158)	0.799	0.742 to 0.857
*Cirrus*	*30*	*2857 (2596)*	*0*.*703*	*0*.*675 to 0*.*731*	**Macula–Total Thickness**				
*Stratus*	*25*	*1732 (1786)*	*0*.*754*	*0*.*729 to 0*.*778*	Average	10	688 (440)	0.792	0.744 to 0.840
*Spectralis*	*11*	*806 (758)*	*0*.*768*	*0*.*724 to 0*.*813*	*Stratus*	*5*	*261 (238)*	*0*.*781*	*0*.*734 to 0*.*829*
*RTVue*	*11*	*894 (821)*	*0*.*762*	*0*.*714 to 0*.*810*	*RTVue*	*3*	*289 (144)*	*0*.*777*	*0*.*656 to 0*.*898*
*Topcon*	*5*	*433 (294)*	*0*.*612*	*0*.*548 to 0*.*676*	Superior Outer				
Temporal	84	6929 (6386)	0.760	0.742 to 0.778	Stratus	4	791 (765)	0.767	0.732 to 0.803
*Cirrus*	*30*	*2857 (2596)*	*0*.*759*	*0*.*729 to 0*.*790*	Inferior Outer				
*Stratus*	*26*	*1793 (1843)*	*0*.*758*	*0*.*729 to 0*.*788*	Stratus	4	791 (765)	0.819	0.786 to 0.851
*Spectralis*	*11*	*806 (857)*	*0*.*759*	*0*.*704 to 0*.*814*	Temporal Outer				
*RTVue*	*12*	*1040 (895)*	*0*.*791*	*0*.*746 to 0*.*835*	Stratus	4	791 (765)	0.773	0.736 to 0.811
*Topcon*	*5*	*433 (294)*	*0*.*707*	*0*.*644 to 0*.*770*	Nasal Outer				
					Stratus	4	791 (765)	0.746	0.695 to 0.796
**Macula—GCIPL**					Superior Inner				
Average	20	1860 (1469)	0.864	0.837 to 0.890	Stratus	4	730 (708)	0.708	0.623 to 0.793
*Cirrus*	*14*	*1308 (1146)*	*0*.*880*	*0*.*851 to 0*.*910*	Inferior Inner				
*Topcon*	*5*	*475 (264)*	*0*.*805*	*0*.*767 to 0*.*843*	Stratus	4	791 (765)	0.755	0.695 to 0.816
Inferior	21	1804 (1547)	0.861	0.836 to 0.886	Temporal Inner				
*Cirrus*	*13*	*1245 (1095)*	*0*.*874*	*0*.*839 to 0*.*908*	Stratus	4	791 (765)	0.742	0.691 to 0.792
*Spectralis*	*2*	*120 (120)*	*0*.*841*	*0*.*791 to 0*.*890*	Nasal Inner				
*Topcon*	*6*	*439 (332)*	*0*.*841*	*0*.*809 to 0*.*872*	Stratus	4	730 (708)	0.670	0.549 to 0.790
Superior	21	1804 (1547)	0.787	0.751 to 0.823					
*Cirrus*	*13*	*1245 (1095)*	*0*.*825*	*0*.*786 to 0*.*864*					
*Spectralis*	*2*	*120 (120)*	*0*.*697*	*0*.*629 to 0*.*765*					
*Topcon*	*6*	*439 (332)*	*0*.*734*	*0*.*693 to 0*.*775*					
Nasal	4	240 (240)	0.647	0.589 to 0.704					
*Spectralis*	*2*	*120 (120)*	*0*.*668*	*0*.*599 to 0*.*737*					
*Topcon*	*2*	*120 (120)*	*0*.*624*	*0*.*534 to 0*.*715*					
Temporal	4	240 (240)	0.811	0.747 to 0.876					
*Spectralis*	*2*	*120 (120)*	*0*.*811*	*0*.*686 to 0*.*936*					
*Topcon*	*2*	*120 (120)*	*0*.*806*	*0*.*753 to 0*.*860*					
Superotemporal	13	1018 (927)	0.835	0.792 to 0.877					
Cirrus	11	835 (827)	0.840	0.793 to 0.887					
Superonasal	13	1018 (927)	0.778	0.727 to 0.829					
Cirrus	11	835 (827)	0.789	0.734 to 0.844					
Inferotemporal	13	1018 (927)	0.879	0.848 to 0.910					
Cirrus	11	835 (827)	0.874	0.838 to 0.909					
Inferonasal	13	1018 (927)	0.789	0.752 to 0.827					
Cirrus	11	835 (827)	0.792	0.745 to 0.838					
Minimum									
Cirrus	10	777 (780)	0.904	0.875 to 0.933					

Average RNFL (0.905, CI95% 0.895 to 0.916), average macular GCIPL (0.864, CI95% 0.837 to 0.890), average macular GCC (0.895, CI95% 0.874 to 0.916) performed similarly well for glaucoma diagnosis. Conversely, average macular mNFL (0.799, CI95% 0.742 to 0.857) and average total macular thickness (0.792, CI95% 0.744 to 0.840) had lower AUROC. Across OCT devices, no major differences were noted for any of the parameters.

#### Pre-perimetric glaucoma

**Fig 5:**
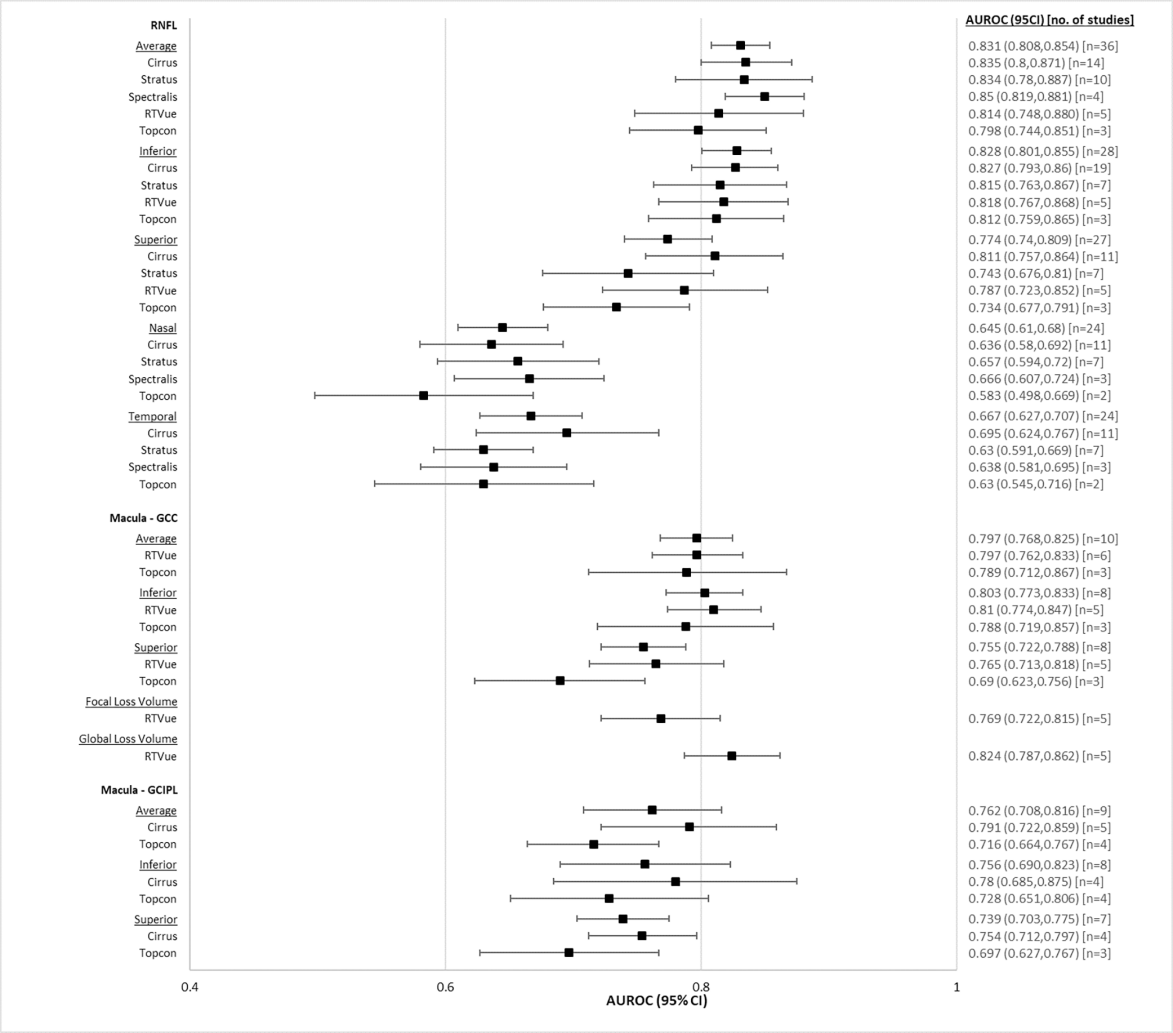
Forest plot of diagnostic accuracies of RNFL and macular OCT parameters, pre-perimetric glaucoma.

Pooled AUROCs for pre-perimetric glaucoma patients are reported in Table 4, and illustrated in a forest plot (Fig 5). There were no major differences across devices for any of the RNFL or macular parameters. Across RNFL parameters, average (0.831, CI95% 0.808 to 0.854), inferior (0.828, CI95% 0.801 to 0.855) and superior (0.774, CI95% 0.740 to 0.809) had larger AUROC than nasal (0.645, CI95% 0.610 to 0.680) or temporal (0.667, CI95% 0.627 to 0.707). All parameters within both macula GCIPL and macula GCC scans performed similarly well.

**Table 4:** Pooled AUROCs of RNFL and macular OCT parameters for pre-perimetric glaucoma patients.

Pre—Perimetric Glaucoma–Pooled AUROCs (if I^2^ > 50% random effects meta-analysis was used, if I^2^ < 50% fixed effects was used)									
Test Parameter, Location and OCT Device	Number of Patient Groups	Pooled Sample Size (controls)	Pooled AUROC	95% CI	Test Parameter, Location and OCT Device	Number of Studies	Pooled Sample Size	Pooled AUROC	95% CI
**RNFL**					**Macula—GCC**				
Average	36	1664 (2541)	0.831	0.808 to 0.854	Average	10	526 (525)	0.797	0.768 to 0.825
Cirrus	14	622 (1186)	0.835	0.800 to 0.871	RTVue	6	365 (333)	0.797	0.762 to 0.833
Stratus	10	399 (565)	0.834	0.780 to 0.887	Topcon	3	112 (141)	0.789	0.712 to 0.867
Spectralis	4	208 (341)	0.850	0.819 to 0.881	Inferior	8	425 (409)	0.803	0.773 to 0.833
RTVue	5	313 (268)	0.814	0.748 to 0.880	RTVue	5	313 (268)	0.81	0.774 to 0.847
Topcon	3	122 (181)	0.798	0.744 to 0.851	Topcon	3	112 (141)	0.788	0.719 to 0.857
Inferior	28	1256 (1748)	0.828	0.801 to 0.855	Superior	8	425 (409)	0.755	0.722 to 0.788
Cirrus	19	834 (1225)	0.827	0.793 to 0.860	RTVue	5	313 (268)	0.765	0.713 to 0.818
Stratus	7	299 (420)	0.815	0.763 to 0.867	Topcon	3	112 (141)	0.69	0.623 to 0.756
RTVue	5	313 (268)	0.818	0.767 to 0.868	Focal Loss Volume				
Topcon	3	122 (181)	0.812	0.759 to 0.865	RTVue	5	249 (281)	0.769	0.722 to 0.815
Superior	27	1256 (1711)	0.774	0.740 to 0.809	Global Loss Volume				
Cirrus	11	487 (770)	0.811	0.757 to 0.864	RTVue	5	249 (281)	0.824	0.787 to 0.862
Stratus	7	299 (420)	0.743	0.676 to 0.810					
RTVue	5	313 (268)	0.787	0.723 to 0.852	**Macula—GCIPL**				
Topcon	3	122 (181)	0.734	0.677 to 0.791	Average	9	395 (732)	0.762	0.708 to 0.816
Nasal	24	1025 (1560)	0.645	0.610 to 0.680	Cirrus	5	205 (487)	0.791	0.722 to 0.859
Cirrus	11	487 (770)	0.636	0.580 to 0.692	Topcon	4	190 (245)	0.716	0.664 to 0.767
Stratus	7	299 (420)	0.657	0.594 to 0.720	Inferior	8	346 (681)	0.756	0.690 to 0.823
Spectralis	3	131 (244)	0.666	0.607 to 0.724	Cirrus	4	156 (436)	0.780	0.685 to 0.875
Topcon	2	82 (106)	0.583	0.498 to 0.669	Topcon	4	190 (245)	0.728	0.651 to 0.806
Temporal	24	1025 (1570)	0.667	0.627 to 0.707	Superior	7	278 (617)	0.739	0.703 to 0.775
Cirrus	11	487 (770)	0.695	0.624 to 0.767	Cirrus	4	156 (436)	0.754	0.712 to 0.797
Stratus	7	299 (420)	0.630	0.591 to 0.669	Topcon	3	122 (181)	0.697	0.627 to 0.767
Spectralis	3	131 (244)	0.638	0.581 to 0.695					
Topcon	2	82 (106)	0.63	0.545 to 0.716					

Overall, average RNFL (0.831, CI95% 0.808 to 0.854) had higher AUROC for glaucoma diagnosis than both average macula GCIPL (0.762, CI95% 0.708 to 0.816) and average macula GCC (0.797, CI95% 0.768 to 0.825).

#### Mild glaucoma

**Fig 6:**
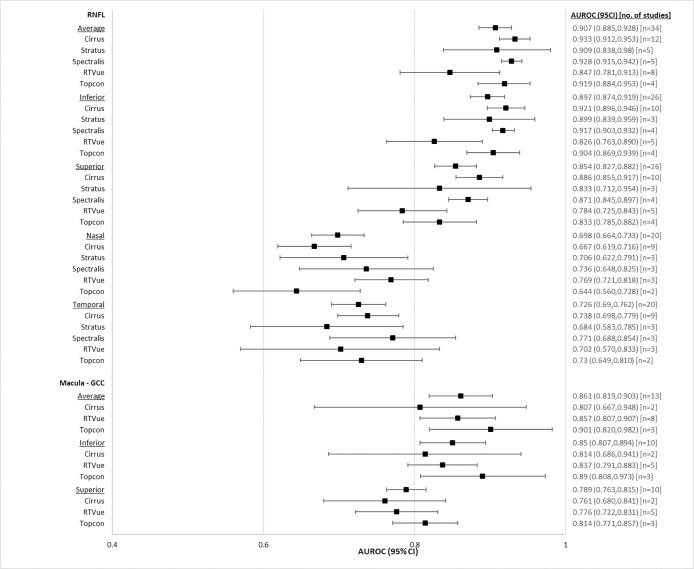
Forest plot of diagnostic accuracies of RNFL and macular OCT parameters, mild glaucoma.

The diagnostic capability of OCT for patients with mild glaucoma is summarized in Table 5, and illustrated in Fig 6. RTVue OCT demonstrated a smaller AUROC than the other reviewed OCT devices for RNFL average (0.847, CI95% 0.781 to 0.913), inferior (0.826, CI95% 0.763 to 0.890), and superior parameters (0.784, CI95% 0.725 to 0.843). Across RNFL parameters, again average (0.912, CI95% 0.892 to 0.932), superior (0.860, CI95% 0.834 to 0.865) and inferior (0.901, CI95% 0.881 to 0.921) areas have higher diagnostic value than nasal (0.700, CI95% 0.667 to 0.732) and temporal (0.732, CI95% 0.698 to 0.766) regions. For macular GCC scans, all areas performed similarly well. Overall, RNFL parameters had higher AUROC than macular GCC (average RNFL (0.912, CI95% 0.892 to 0.932), average macular GCC (0.861, CI95% 0.819 to 0.903)).

**Table 5:** Pooled AUROCs of RNFL and macular OCT parameters for mild glaucoma patients.

Mild Glaucoma–Pooled AUROCs (if I^2^ > 50% random effects meta-analysis was used, if I^2^ < 50% fixed effects was used)									
Test Parameter, Location and OCT Device	Number of Studies	Pooled Sample Size	Pooled AUROC	95% CI	Test Parameter, Location and OCT Device	Number of Studies	Pooled Sample Size	Pooled AUROC	95% CI
**RNFL**					**Macula—GCC**				
Average	34	2146 (2782)	0.907	0.885 to 0.928	Average	13	817 (836)	0.861	0.819 to 0.903
Cirrus	12	990 (1409)	0.933	0.912 to 0.953	Cirrus	2	143 (128)	0.807	0.667 to 0.948
Stratus	5	225 (308)	0.909	0.838 to 0.980	RTVue	8	467 (540)	0.857	0.807 to 0.907
Spectralis	5	193 (282)	0.928	0.915 to 0.942	Topcon	3	207 (168)	0.901	0.820 to 0.982
RTVue	8	467 (540)	0.847	0.781 to 0.913					
Topcon	4	271 (243)	0.919	0.884 to 0.953	Inferior	10	686 (721)	0.850	0.807 to 0.894
					Cirrus	2	143 (128)	0.814	0.686 to 0.941
Inferior	26	1724 (2393)	0.897	0.874 to 0.919	RTVue	5	336 (425)	0.837	0.791 to 0.883
Cirrus	10	808 (1253)	0.921	0.896 to 0.946	Topcon	3	207 (168)	0.890	0.808 to 0.973
Stratus	3	171 (232)	0.899	0.839 to 0.959					
Spectralis	4	138 (240)	0.917	0.903 to 0.932	Superior	10	686 (721)	0.789	0.763 to 0.815
RTVue	5	336 (425)	0.826	0.763 to 0.890	Cirrus	2	143 (128)	0.761	0.680 to 0.841
Topcon	4	271 (243)	0.904	0.869 to 0.939	RTVue	5	336 (425)	0.776	0.722 to 0.831
					Topcon	3	207 (168)	0.814	0.771 to 0.857
Superior	26	1720 (2393)	0.854	0.827 to 0.882					
Cirrus	10	808 (1253)	0.886	0.855 to 0.917					
Stratus	3	167 (232)	0.833	0.712 to 0.954					
Spectralis	4	138 (240)	0.871	0.845 to 0.897					
RTVue	5	336 (425)	0.784	0.725 to 0.843					
Topcon	4	271 (243)	0.833	0.785 to 0.882					
Nasal	20	1302 (1549)	0.698	0.664 to 0.733					
Cirrus	9	700 (745)	0.667	0.619 to 0.716					
Stratus	3	171 (232)	0.706	0.622 to 0.791					
Spectralis	3	88 (190)	0.736	0.648 to 0.825					
RTVue	3	200 (254)	0.769	0.721 to 0.818					
Topcon	2	143 (128)	0.644	0.560 to 0.728					
Temporal	20	1302 (1549)	0.726	0.690 to 0.762					
Cirrus	9	700 (745)	0.738	0.698 to 0.779					
Stratus	3	171 (232)	0.684	0.583 to 0.785					
Spectralis	3	88 (190)	0.771	0.688 to 0.854					
RTVue	3	200 (254)	0.702	0.570 to 0.833					
Topcon	2	143 (128)	0.730	0.649 to 0.810					

#### Moderate to severe glaucoma

**Fig 7:**
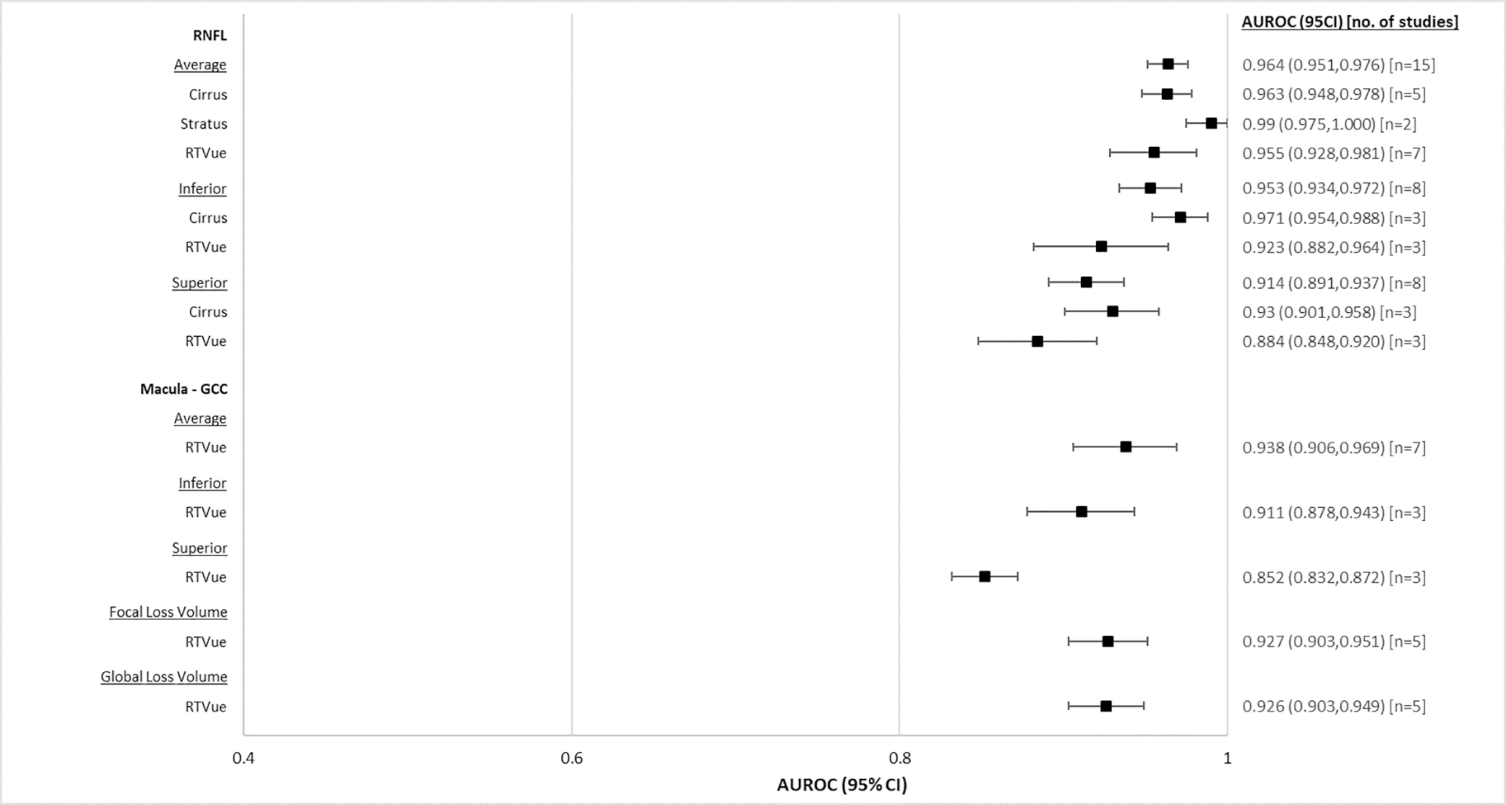
Forest plot of diagnostic accuracies of RNFL and macular OCT parameters, moderate to severe glaucoma.

AUROCs of OCT for patients with moderate to severe glaucoma are summarized in Table 6, and illustrated in Fig 7. Overall, all OCT devices performed similarly well for glaucoma diagnosis. All RNFL parameters reported—average (0.959, CI95% 0.946 to 0.972), superior (0.923, CI95% 0.905 to 0.941) and inferior (0.954, CI95% 0.935 to 0.972)–had similar AUROCs. Superior macular GCC (0.856, CI95% 0.837 to 0.876), performed worse than the remainder of the macular GCC parameters. RNFL and macular GCC have comparable AUROCs (average RNFL (0.959, CI95% 0.946 to 0.972), macula GCC (0.938, CI95% 0.911 to 0.965)).

**Table 6:** Pooled AUROCs of RNFL and macular OCT parameters for moderate to severe glaucoma patients.

Moderate to Severe Glaucoma–Pooled AUROCs (if I^2^ > 50% random effects meta-analysis was used, if I^2^ < 50% fixed effects was used)									
Test Parameter, Location and OCT Device	Number of Studies	Pooled Sample Size	Pooled AUROC	95% CI	Test Parameter, Location and OCT Device	Number of Studies	Pooled Sample Size	Pooled AUROC	95% CI
**RNFL**					**Macula—GCC**				
Average	15	752 (1465)	0.964	0.951 to 0.976	Average				
Cirrus	5	353 (857)	0.963	0.948 to 0.978	RTVue	7	299 (434)	0.938	0.906 to 0.969
Stratus	2	74 (109)	0.990	0.975 to 1.000					
RTVue	7	299 (434)	0.955	0.928 to 0.981	Inferior				
					RTVue	3	163 (274)	0.911	0.878 to 0.943
Inferior	8	485 (1114)	0.953	0.934 to 0.972					
Cirrus	3	248 (701)	0.971	0.954 to 0.988	Superior				
RTVue	3	163 (274)	0.923	0.882 to 0.964	RTVue	3	163 (204)	0.852	0.832 to 0.872
Superior	8	485 (1114)	0.914	0.891 to 0.937	Focal Loss Volume				
Cirrus	3	248 (701)	0.930	0.901 to 0.958	RTVue	5	240 (364)	0.927	0.903 to 0.951
RTVue	3	163 (274)	0.884	0.848 to 0.920					
					Global Loss Volume				
					RTVue	5	240 (364)	0.926	0.903 to 0.949

#### Myopic patients

**Fig 8:**
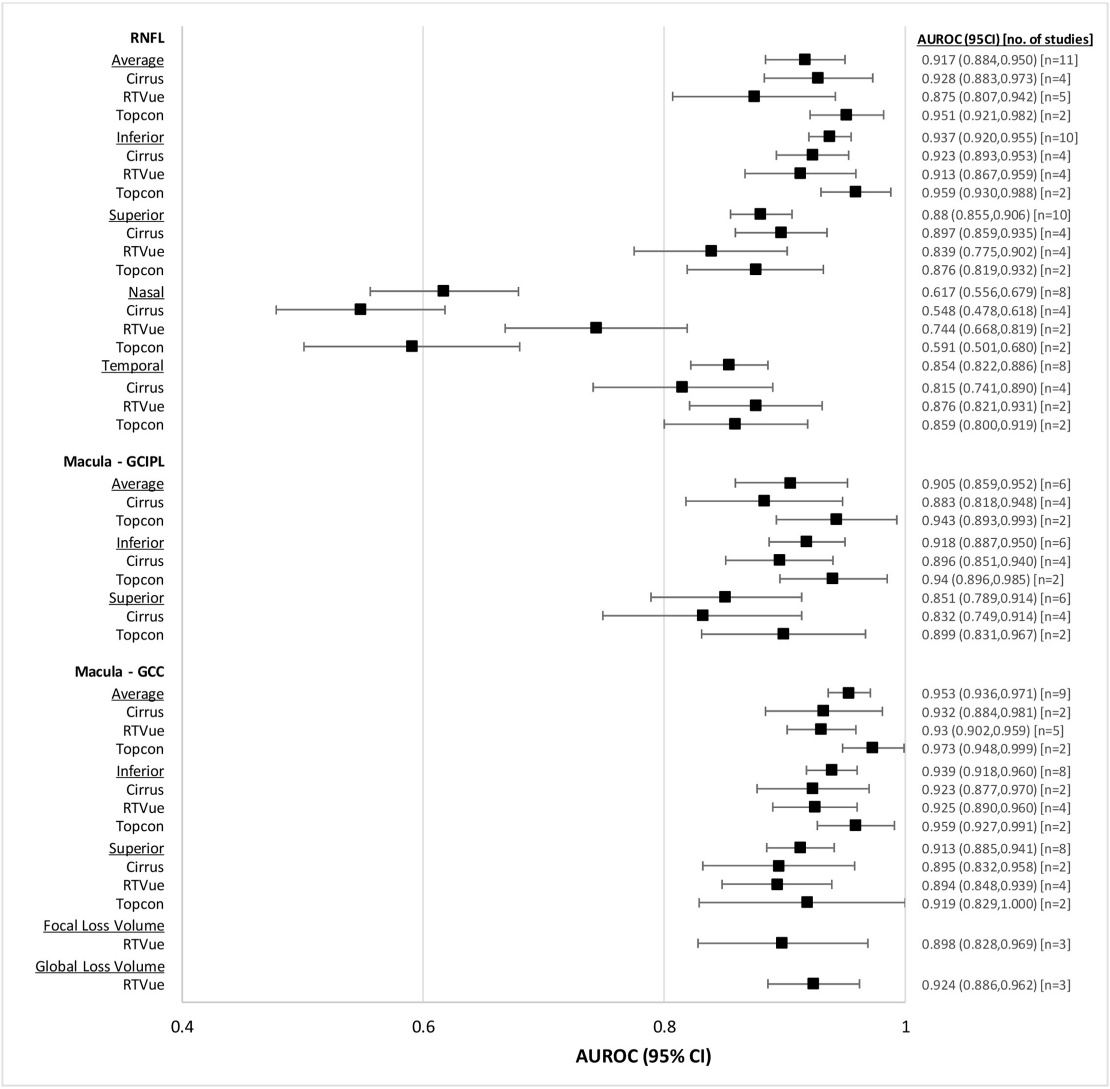
Forest plot of diagnostic accuracies of RNFL and macular OCT parameters, myopic patients.

AUROCs of OCT for glaucoma diagnosis in myopic patients are summarized in Table 7, and illustrated in Fig 8. All OCT devices performed relatively similarly for glaucoma diagnosis. Within RNFL, the average (0.917, CI95% 0.884 to 0.950), inferior (0.937, CI95% 0.920 to 0.955), superior (0.880, CI95% 0.855 to 0.906), and temporal (0.854, CI95% 0.822 to 0.886) parameters had improved AUROC compared to the nasal area (0.617, CI95% 0.556 to 0.679). For both macular GCIPL and macular GCC scans, diagnostic performance of all individual parameters was similar. In addition, there were no notable differences in AUROC for the average parameters of RNFL (0.917, CI95% 0.884 to 0.950), macular GCIPL (0.905, CI95% 0.859 to 0.952), and macular GCC (0.953, CI95% 0.936 to 0.971) scans.

**Table 7:** Pooled AUROCs of RNFL and macular OCT parameters for myopic patients.

Myopic Patients–Pooled AUROCs (if I^2^ > 50% random effects meta-analysis was used, if I^2^ < 50% fixed effects was used)									
Test Parameter, Location and OCT Device	Number of Studies	Pooled Sample Size	Pooled AUROC	95% CI	Test Parameter, Location and OCT Device	Number of Studies	Pooled Sample Size	Pooled AUROC	95% CI
**RNFL**					**Macula—GCC**				
Average	11	586 (461)	0.917	0.884 to 0.950	Average	9	509 (411)	0.953	0.936 to 0.971
Cirrus	4	213 (157)	0.928	0.883 to 0.973	Cirrus	2	136 (107)	0.932	0.884 to 0.981
RTVue	5	237 (197)	0.875	0.807 to 0.942	RTVue	5	237 (197)	0.930	0.902 to 0.959
Topcon	2	136 (107)	0.951	0.921 to 0.982	Topcon	2	136 (107)	0.973	0.948 to 0.999
Inferior	10	534 (423)	0.937	0.920 to 0.955	Inferior	8	457 (373)	0.939	0.918 to 0.960
Cirrus	4	213 (157)	0.923	0.893 to 0.953	Cirrus	2	136 (107)	0.923	0.877 to 0.970
RTVue	4	185 (159)	0.913	0.867 to 0.959	RTVue	4	185 (159)	0.925	0.890 to 0.960
Topcon	2	136 (107)	0.959	0.930 to 0.988	Topcon	2	136 (107)	0.959	0.927 to 0.991
Superior	10	534 (423)	0.880	0.855 to 0.906	Superior	8	457 (313)	0.913	0.885 to 0.941
Cirrus	4	213 (157)	0.897	0.859 to 0.935	Cirrus	2	136 (107)	0.895	0.832 to 0.958
RTVue	4	185 (159)	0.839	0.775 to 0.902	RTVue	4	185 (159)	0.894	0.848 to 0.939
Topcon	2	136 (107)	0.876	0.819 to 0.932	Topcon	2	136 (107)	0.919	0.829 to 1.000
Nasal	8	485 (371)	0.617	0.556 to 0.679	Focal Loss Volume				
Cirrus	4	213 (157)	0.548	0.478 to 0.618	RTVue	3	101 (90)	0.898	0.828 to 0.969
RTVue	2	136 (107)	0.744	0.668 to 0.819					
Topcon	2	136 (107)	0.591	0.501 to 0.680	Global Loss Volume				
					RTVue	3	101 (90)	0.924	0.886 to 0.962
Temporal	8	485 (371)	0.854	0.822 to 0.886					
Cirrus	4	213 (157)	0.815	0.741 to 0.890					
RTVue	2	136 (107)	0.876	0.821 to 0.931					
Topcon	2	136 (107)	0.859	0.800 to 0.919					
**Macula—GCIPL**									
Average	6	349 (264)	0.905	0.859 to 0.952					
Cirrus	4	213 (157)	0.883	0.818 to 0.948					
Topcon	2	136 (107)	0.943	0.893 to 0.993					
Inferior	6	349 (264)	0.918	0.887 to 0.950					
Cirrus	4	213 (157)	0.896	0.851 to 0.940					
Topcon	2	136 (107)	0.940	0.896 to 0.985					
Superior	6	349 (264)	0.851	0.789 to 0.914					
Cirrus	4	213 (157)	0.832	0.749 to 0.914					
Topcon	2	136 (107)	0.899	0.831 to 0.967					

### Evaluation of publication bias

Funnel plots were constructed to evaluate publication bias in the meta-analysis. Several funnel plots were created, one for each imaging parameter (average, superior, inferior etc.), of each area (RNFL, macula), for each OCT device, within each patient subgroup. No pattern was evident, ie. no one patient group, OCT device, or scan type/parameter was noted to be more likely to have publication bias.

## Discussion

This meta-analysis demonstrates that OCT is a valuable adjunctive tool to aid in glaucoma diagnosis. Pooled estimates of diagnostic accuracy (AUROC) for the most commonly used OCT instruments (Zeiss Cirrus OCT, Zeiss, Stratus OCT, Heidelberg Spectralis, Optovue RTVue, Topcon 3D-OCT) were determined based upon their ability to differentiate between normal participants and glaucoma patients. A summary of the technical features of each device are outlined in Table 8.

**Table 8:** Technical features of each of the OCT devices studied [27,180].

Model	Zeiss Stratus	Zeiss Cirrus	Heidelberg Spectralis	Optovue RTVue	Topcon 3D-OCT
**Key Features**	- Sequential acquisition	- Simultaneous acquisition			
	- 1 pixel at a time	- Entire A-scan collected at once			
	- Utilizes a mirror	- Faster than eye movements			
		- Does not utilize a mirror			
		- Analyzes data using a spectrometer			
**Scanning Speed (A-scans/sec)**	400	27,000–68,000	40,000	70,000	27,000
**Axial resolution (microns)**	10	5	3.9	5	5–6
**Imaging modes available**	TD-OCT	SD-OCT	SD-OCT	SD-OCT	SD-OCT
		cLSO	IR fundus photo with cLSO		
**Scanning range**	Retina/nerve	Retina/nerve	Retina/nerve	Retina/nerve	Retina/nerve
		Anterior segment		Cornea	Anterior segment
				Angle	
**Posterior Segment Analyses**	Macula: Total thickness	Macula: Macular thickness, macular changes, ganglion cells, RPE changes	Macula: Real-time, fast, dense, detail, posterior pole, seven lines	Macula: Retinal trend analysis, ganglion cell complex, retinal overview report, multilayers en face report	Macula: 3D macula report, macular drusen analysis
	Nerve: RNFL thickness	Nerve: RNFL thickness, guided progression	Nerve: Fast, dense, posterior pole, nerve head circle	Nerve: Retinal nerve fiber and optic disc, optic disc structure and analysis	Nerve: 3D disc report, RNFL trend analysis, glaucoma analysis
		3D imaging		Wide-field en face mapping	Glaucoma and macula report (12 × 9 mm)
				Combined RNFL and ganglion cell change report	

cLSO: confocal laser scanning ophthalmoscope; TD-OCT: time domain optical coherence tomography; SD-OCT: spectral domain optical coherence tomography; RNFL: retinal nerve fiber layer

The 150 studies included reported the diagnostic capability of several RNFL and macular parameters. Macular scans were further subdivided by retinal segmentation (GCC, GCIPL, mNFL or total retinal thickness). The AUROCs for average, superior and inferior RNFL parameters were larger than for nasal and temporal areas, a finding that was consistent for the overall patient group, as well as glaucoma subgroups. This finding is explained by the work of Traynis et al., 2014 who proposed a schematic of glaucomatous damage to the macula. Retinal ganglion cells (RCGs) in the regions of the macula most vulnerable to glaucomatous damage (inferior macula and region outside of the central 8 degrees of macula), project to the inferior and superior quadrants of the optic disc. Whereas RCGs in the less vulnerable regions (superior macula), project to the temporal region of the disc [179].

By comparison, in the macular GCIPL scans, we found that the inferonasal and superonasal parameters had poorer diagnostic efficacy than the average, superior, inferior, and temporal (infero- and superotemporal parameters). These differences between parameters were not found in the macular GCC scans.

Comparing between different scan types, RNFL thickness, macular GCIPL and macular GCC had similar diagnostic capability to differentiate between normal and glaucomatous eyes. Total macular thickness had lower AUROC for glaucoma diagnosis than these more specific scan types. Through stratification of patients by disease severity for sub-analysis, we also note that the diagnostic capability of OCT improves with increased disease severity.

One major question we wished to address through this review was whether there were instrument-dependent differences in diagnostic ability of OCT. It appears that for the majority of subgroups, there are no notable differences between devices.

### Comparison with other reviews

Previous reviews on the diagnostic capability of OCT for glaucoma have been published[14,181–185]. The present review has some unique advantages over previous reports. First, as mentioned previously, a wide gold standard was accepted for inclusion, ie. White-on-white standard automated perimetry, optic disc appearance, elevated IOP, or any combination thereof. This wide gold standard more accurately reflects true clinical practice, where patients undergoing OCT to aid in glaucoma diagnosis may have undergone many of these other diagnostic modalities previously. The majority of previous reviews have limited inclusion criteria to those patients who have exclusively undergone standard automated perimetry. Our approach enabled the inclusion of 150 OCT studies, markedly larger than previous meta-analyses; a Cochrane review by Michelessi et al.[181] identified 63 OCT studies, Fallon et al.[185] identified 47 studies, Ahmed et al.[182] identified 84 studies, and Oddone et al.[183] identified 34 studies. The larger number of studies included enabled a more robust meta-analysis and the analyses of several patient subgroups.

Importantly, this meta-analysis provides pooled estimates of AUROCs, rather than sensitivity and specificity, as used in previous reviews. Only one other OCT review, by Chen et al.[184] identified reported pooled AUROCs; however, that review was limited to only 21 studies of Zeiss Stratus OCT. Reporting of AUROC is advantageous when describing the utility of a diagnostic test as it represents the diagnostic capability of the test regardless of specific cutoff used. We found that individual studies were inconstant in their reporting of sensitivity and specificity, with certain studies reporting sensitivities and particular specificity cutoffs, and others reporting the “optimal” sensitivity/specificity cutoff. Meta-analysis of such inconsistent data is difficult.

### Limitations

One limitation of this study was the relatively large number of case-control studies that were captured in the inclusion criteria. The case-control design has been suggested to overestimate accuracy[186]. As the main purpose was to compare the diagnostic performance of the most common currently used OCT devices and none were found to be superior, this limitation unlikely introduced any significant bias. Another limitation may have resulted from choosing to the compare a number of macular parameters. Unlike RNFL scans, studies were quite heterogeneous in terms of which macular parameters were reported, ie. some reported GCIPL, GCC, mNFL, and total thickness. As such, these scan types had to be separated for meta-analysis, reducing sample sizes, and consequently increasing instability of AUROC estimates. Importantly, all studies included in the meta-analysis evaluated the ability to differentiate healthy controls from confirmed glaucoma patients, which does not reflect real clinical practice where many patients are undifferentiated.

## Conclusion

The currently available OCT devices (Zeiss Cirrus, Zeiss Stratus, Heidelberg Spectralis, Optovue RTVue, Topcon 3D-OCT) demonstrated good diagnostic accuracy in their ability to differentiate glaucoma patients from normal controls. This ability increased with the severity of the glaucoma. There was no major device-related differences in diagnostic capacity. Within RNFL scans, the nasal and temporal parameters are more poorly diagnostic than the average, superior and inferior parameters. Across all macular GCIPL scans, the nasal (supero- and infero-nasal) parameters had lower AUROCs than the average, superior, inferior and temporal regions. The diagnostic capacity of RNFL is similar to segmented macular regions (GCIPL, GCC), and better than total macular thickness. As OCT technology continues to evolve at a faster pace than functional assessments of optic nerve health, future studies will be needed to fully understand its role in glaucoma management.

## Supporting information

S1 Table Appendix 1 –Search strategies. (PDF)

S2 Table Appendix 2 –Individual study characteristics. (PDF)

S3 Table PRISMA Checklist. (DOC)
