# Supplementary material for: Optical coherence tomography for glaucoma diagnosis: An evidence based meta-analysis
Source: PLoS One. 2018 Jan 4;13(1):e0190621. doi: 10.1371/journal.pone.0190621 (PMC5754143; doi:10.1371/journal.pone.0190621)
Supplement: S1 Table — (PDF) [file pone.0190621.s001.pdf]

Appendix 1: Search strategies for each OCT device, for each database searched.

Zeiss Search Strategy:

MEDLINE (OVID) Search Strategy:

	Search Term	Results
1	exp glaucoma/	50110
2	Glaucoma*.mp.	65438
3	zeiss.mp.	2930
4	cirrus.mp. or stratus.mp.	2361
5	exp Tomography/ or exp Tomography, Optical Coherence/ or oct.mp.	890213
6	sensitivity.mp. or exp "Sensitivity and Specificity"/	1235701
7	specificity.mp. or exp "Sensitivity and Specificity"/	1226779
8	exp "Predictive Value of Tests"/ or positive predictive value.mp.	214267
9	exp "Predictive Value of Tests"/ or negative predictive value.mp.	209916
10	exp ROC Curve/ or ROC.mp. or (receiver-operating characteristic).mp.	84300
11	"Sensitivity and Specificity"/ or diagnostic odds ratio.mp.	346085
12	Likelihood Functions/ or likelihood ratio.mp.	30542
13	(1 or 2) and (3 or 4) and 5 and (6 or 7 or 8 or 9 or 10 or 11 or 12)	**282**

EMBASE (OVID) Search Strategy:

	Search Term	Results
1	exp glaucoma/	79920
2	Glaucoma*.mp.	83206
3	zeiss.mp.	8005
4	cirrus.mp. or stratus.mp.	3274
5	exp Tomography/ or exp Tomography, Optical Coherence/ or oct.mp.	933476
6	sensitivity.mp. or exp "Sensitivity and Specificity"/	1183992
7	specificity.mp. or exp "Sensitivity and Specificity"/	746788
8	exp "Predictive Value of Tests"/ or positive predictive value.mp.	135572
9	exp "Predictive Value of Tests"/ or negative predictive value.mp.	130686
10	exp ROC Curve/ or ROC.mp. or (receiver-operating characteristic).mp.	108047
11	"Sensitivity and Specificity"/ or diagnostic odds ratio.mp.	273864
12	Likelihood Functions/ or likelihood ratio.mp.	166297
13	(1 or 2) and (3 or 4) and 5 and (6 or 7 or 8 or 9 or 10 or 11 or 12)	**360**

CINAHL Search Strategy:

	Search Term	Results
1	(MH "Glaucoma+")	3315
2	Glaucoma*	3709
3	"zeiss"	33
4	“cirrus”	12

5	"stratus"	16
6	(s1 or s2) and (s3 or s4 or s5)	**4**

Cochrane Library Search Strategy:

	Search Term	Results
1	MeSH descriptor: [Glaucoma] explode all trees	2427
2	glaucoma*:ti,ab,kw (Word variations have been searched)	5231
3	zeiss:ti,ab,kw	220
4	cirrus:ti,ab,kw	42
5	stratus:ti,ab,kw	98
6	(#1 or #2) and (#3 or #4 or #5)	**65**

Web of Science Search Strategy:

	Search Term	Results
1	TS=(glaucoma*)	49,859
2	TS=(zeiss)	2899
3	TS=(cirrus OR stratus)	9769
4	TS=(oct OR optical coherence tomography OR tomography)	418696
5	TS=(sensitivity OR specificity OR positive predictive value OR negative predictive value OR ROC OR diagnostic odds ratio OR likelihood ratio)	1353033
6	#1 and (#2 or #3) and #4 and #5	**223**

BIOSIS Search Strategy:

	Search Term	Results
1	TS=(glaucoma*)	49580
2	TS=(zeiss)	2871
3	TS=(cirrus)	7198
4	TS=(stratus)	2864
5	TS=(oct OR optical coherence tomography OR tomography)	418628
6	TS=(sensitivity OR specificity OR positive predictive value OR negative predictive value OR ROC OR diagnostic odds ratio OR likelihood ratio)	1350893
7	#1 and (#2 or #3 or #4) and #5 and #6	**28**

Heidelberg Search Strategy:

MEDLINE (OVID) Search Strategy:

	Search Term	Results
1	exp glaucoma/	50135
2	Glaucoma*.mp.	65492
3	heidelberg.mp.	5089
4	spectralis.mp.	705
5	exp Tomography/ or exp Tomography, Optical Coherence/ or oct.mp.	891961
6	sensitivity.mp. or exp "Sensitivity and Specificity"/	1237771
7	specificity.mp. or exp "Sensitivity and Specificity"/	1226472
8	exp "Predictive Value of Tests"/ or positive predictive value.mp.	214689
9	exp "Predictive Value of Tests"/ or negative predictive value.mp.	210325
10	exp ROC Curve/ or ROC.mp. or (receiver-operating characteristic).mp.	84516
11	"Sensitivity and Specificity"/ or diagnostic odds ratio.mp.	346843
12	Likelihood Functions/ or likelihood ratio.mp.	30579
13	(1 or 2) and (3 or 4) and 5 and (6 or 7 or 8 or 9 or 10 or 11 or 12)	**281**

EMBASE (OVID) Search Strategy:

	Search Term	Results
1	exp glaucoma/	79984
2	Glaucoma*.mp.	83268
3	heidelberg.mp.	38816
4	spectralis.mp.	1694
5	exp Tomography/ or exp Tomography, Optical Coherence/ or oct.mp.	934735
6	sensitivity.mp. or exp "Sensitivity and Specificity"/	1185110
7	specificity.mp. or exp "Sensitivity and Specificity"/	747443
8	exp "Predictive Value of Tests"/ or positive predictive value.mp.	135863
9	exp "Predictive Value of Tests"/ or negative predictive value.mp.	130975
10	exp ROC Curve/ or ROC.mp. or (receiver-operating characteristic).mp.	108280
11	"Sensitivity and Specificity"/ or diagnostic odds ratio.mp.	274210
12	Likelihood Functions/ or likelihood ratio.mp.	166797
13	(1 or 2) and (3 or 4) and 5 and (6 or 7 or 8 or 9 or 10 or 11 or 12)	**275**

CINAHL Search Strategy:

	Search Term	Results
1	(MH "Glaucoma+")	3318
2	Glaucoma*	3713
3	"heidelberg"	179
4	“spectralis”	6
5	(s1 or s2) and (s3 or s4)	**5**

Cochrane Library Search Strategy:

	Search Term	Results
1	MeSH descriptor: [Glaucoma] explode all trees	2427
2	glaucoma*:ti,ab,kw (Word variations have been searched)	5231
3	heidelberg:ti,ab,kw	1012
4	spectralis:ti,ab,kw	37
5	(#1 or #2) and (#3 or #4)	**101**

Web of Science Search Strategy:

	Search Term	Results
1	TS=(glaucoma*)	49,909
2	TS=(heidelberg)	6956
3	TS=(spectralis)	542
4	TS=(oct OR optical coherence tomography OR tomography)	419454
5	TS=(sensitivity OR specificity OR positive predictive value OR negative predictive value OR ROC OR diagnostic odds ratio OR likelihood ratio)	1355232
6	#1 and (#2 or #3) and #4 and #5	**201**

BIOSIS Search Strategy:

	Search Term	Results
1	TS=(glaucoma*)	39,970
2	TS=(heidelbeg)	1
3	TS=(spectralis)	737
4	TS=(oct OR optical coherence tomography OR tomography)	325,536
5	TS=(sensitivity OR specificity OR positive predictive value OR negative predictive value OR ROC OR diagnostic odds ratio OR likelihood ratio)	919,772
6	#1 and (#2 or #3) and #4 and #5	**35**

RTVue Search Strategy:

MEDLINE (OVID) Search Strategy:

	Search Term	Results
1	exp glaucoma/	50145
2	Glaucoma*.mp.	65535
3	RTVue.mp.	369
4	optovue.mp. or (Fourier-domain).mp.	1674
5	exp Tomography/ or exp Tomography, Optical Coherence/ or oct.mp.	892731
6	sensitivity.mp. or exp "Sensitivity and Specificity"/	1239062
7	specificity.mp. or exp "Sensitivity and Specificity"/	1229578
8	exp "Predictive Value of Tests"/ or positive predictive value.mp.	215224
9	exp "Predictive Value of Tests"/ or negative predictive value.mp.	210863
10	exp ROC Curve/ or ROC.mp. or (receiver-operating characteristic).mp.	84664
11	"Sensitivity and Specificity"/ or diagnostic odds ratio.mp.	347092
12	Likelihood Functions/ or likelihood ratio.mp.	30609
13	(1 or 2) and (3 or 4) and 5 and (6 or 7 or 8 or 9 or 10 or 11 or 12)	**93**

EMBASE (OVID) Search Strategy:

	Search Term	Results
1	exp glaucoma/	80004
2	Glaucoma*.mp.	83290
3	RTVue.mp.	613
4	optovue.mp. or (Fourier-domain).mp.	1657
5	exp Tomography/ or exp Tomography, Optical Coherence/ or oct.mp.	935462
6	sensitivity.mp. or exp "Sensitivity and Specificity"/	1185702
7	specificity.mp. or exp "Sensitivity and Specificity"/	747760
8	exp "Predictive Value of Tests"/ or positive predictive value.mp.	135987
9	exp "Predictive Value of Tests"/ or negative predictive value.mp.	131099
10	exp ROC Curve/ or ROC.mp. or (receiver-operating characteristic).mp.	108395
11	"Sensitivity and Specificity"/ or diagnostic odds ratio.mp.	274341
12	Likelihood Functions/ or likelihood ratio.mp.	167095
13	(1 or 2) and (3 or 4) and 5 and (6 or 7 or 8 or 9 or 10 or 11 or 12)	**99**

CINAHL Search Strategy:

	Search Term	Results
1	(MH "Glaucoma+")	3318
2	Glaucoma*	3713
3	"RTVue"	4
4	“optovue” or “fourier-domain”	15
5	(s1 or s2) and (s3 or s4)	**3**

Cochrane Library Search Strategy:

	Search Term	Results
1	MeSH descriptor: [Glaucoma] explode all trees	2427
2	glaucoma*:ti,ab,kw (Word variations have been searched)	5231
3	RTVue:ti,ab,kw	23
4	optovue:ti,ab,kw	7
5	Fourier-domain:ti,ab,kw	43
6	(#1 or #2) and (#3 or #4 or #5)	**12**

Web of Science Search Strategy:

	Search Term	Results
1	TS=(glaucoma*)	49,909
2	TS=(RTVue)	275
3	TS=(optovue OR fourier-domain)	3895
4	TS=(oct OR optical coherence tomography OR tomography)	419453
5	TS=(sensitivity OR specificity OR positive predictive value OR negative predictive value OR ROC OR diagnostic odds ratio OR likelihood ratio)	1355227
6	#1 and (#2 or #3) and #4 and #5	**66**

BIOSIS Search Strategy:

	Search Term	Results
1	TS=(glaucoma*)	39,970
2	TS=(RTVue)	226
3	TS=(optovue OR fourier-domain)	696
4	TS=(oct OR optical coherence tomography OR tomography)	325,536
5	TS=(sensitivity OR specificity OR positive predictive value OR negative predictive value OR ROC OR diagnostic odds ratio OR likelihood ratio)	919,772
6	#1 and (#2 or #3) and #4 and #5	**34**

Topcon Search Strategy:

MEDLINE (OVID) Search Strategy:

	Search Term	Results
1	exp glaucoma/	50175
2	Glaucoma*.mp.	65589
3	topcon.mp.	609
4	oct-2000.mp. or oct-1.mp. or maestro.mp. or triton.mp. or DRI.mp.	26149
5	exp Tomography/ or exp Tomography, Optical Coherence/ or oct.mp.	893875
6	sensitivity.mp. or exp "Sensitivity and Specificity"/	1240764
7	specificity.mp. or exp "Sensitivity and Specificity"/	1231071
8	exp "Predictive Value of Tests"/ or positive predictive value.mp.	215643
9	exp "Predictive Value of Tests"/ or negative predictive value.mp.	211275
10	exp ROC Curve/ or ROC.mp. or (receiver-operating characteristic).mp.	84910
11	"Sensitivity and Specificity"/ or diagnostic odds ratio.mp.	347365
12	Likelihood Functions/ or likelihood ratio.mp.	30641
13	(1 or 2) and (3 or 4) and 5 and (6 or 7 or 8 or 9 or 10 or 11 or 12)	**15**

EMBASE (OVID) Search Strategy:

	Search Term	Results
1	exp glaucoma/	80073
2	Glaucoma*.mp.	83356
3	topcon.mp.	1522
4	oct-2000.mp. or oct-1.mp. or maestro.mp. or triton.mp. or DRI.mp.	28765
5	exp Tomography/ or exp Tomography, Optical Coherence/ or oct.mp.	936633
6	sensitivity.mp. or exp "Sensitivity and Specificity"/	1187301
7	specificity.mp. or exp "Sensitivity and Specificity"/	746706
8	exp "Predictive Value of Tests"/ or positive predictive value.mp.	136367
9	exp "Predictive Value of Tests"/ or negative predictive value.mp.	131479
10	exp ROC Curve/ or ROC.mp. or (receiver-operating characteristic).mp.	108718
11	"Sensitivity and Specificity"/ or diagnostic odds ratio.mp.	274788
12	Likelihood Functions/ or likelihood ratio.mp.	167663
13	(1 or 2) and (3 or 4) and 5 and (6 or 7 or 8 or 9 or 10 or 11 or 12)	**50**

CINAHL Search Strategy:

	Search Term	Results
1	(MH "Glaucoma+")	3328
2	Glaucoma*	3724
3	"topcon"	8
4	“oct-2000” or “oct-1”	293
5	“maestro” or “triton” or “DRI”	220
6	(s1 or s2) and (s3 or s4 or s5)	**0**

Cochrane Library Search Strategy:

	**Search Term**	**Results**
1	MeSH descriptor: [Glaucoma] explode all trees	2427
2	glaucoma*:ti,ab,kw (Word variations have been searched)	5231
3	topcon:ti,ab,kw	80
4	oct-2000:ti,ab,kw	106
5	oct-1:ti,ab,kw	103
6	maestro:ti,ab,kw	23
7	triton:ti,ab,kw	75
8	DRI:ti,ab,kw	87
9	(#1 or #2) and (#3 or #4 or #5 or #6 or #7 or #8)	**13**

Web of Science Search Strategy:

	**Search Term**	**Results**
1	TS=(glaucoma*)	49,948
2	TS=(topcon)	548
3	TS=(oct-2000 OR oct-1)	2220
4	TS=(maestro OR triton OR DRI)	24622
5	TS=(oct OR optical coherence tomography OR tomography)	420069
6	TS=(sensitivity OR specificity OR positive predictive value OR negative predictive value OR ROC OR diagnostic odds ratio OR likelihood ratio)	1357413
7	#1 and (#2 or #3 or #4) and #5 and #6	**10**

BIOSIS Search Strategy:

	**Search Term**	**Results**
1	TS=(glaucoma*)	39,970
2	TS=(topcon)	536
3	TS=(oct-2000 OR oct-1)	4539
4	TS=(maestro OR triton OR DRI)	26,521
5	TS=(oct OR optical coherence tomography OR tomography)	325,536
6	TS=(sensitivity OR specificity OR positive predictive value OR negative predictive value OR ROC OR diagnostic odds ratio OR likelihood ratio)	919,772
7	#1 and (#2 or #3 or #4) and #5 and #6	**9**
