# Supplementary material for: Optical coherence tomography for glaucoma diagnosis: An evidence based meta-analysis
Source: PLoS One. 2018 Jan 4;13(1):e0190621. doi: 10.1371/journal.pone.0190621 (PMC5754143; doi:10.1371/journal.pone.0190621)
Supplement: S2 Table — (PDF) [file pone.0190621.s002.pdf]

Appendix 2: Characteristics of included studies.

Study #	Author	Year	# cases (controls)	Glaucoma Type	Reference Standard	OCT Model	Area Imaged	Country	Study Period (months)	Study Design
1	Ajtony	2007	164<br>(45)	Perimetric	VF+Disc appearance	Stratus	RNFL	Hungary	10	Cross-sectional<br>Retrospective
2	Akashi	2013	145<br>75<br>(87)	Perimetric<br>Mild	VF+Disc appearance	Cirrus<br>RTVue<br>3DTopcon	RNFL+<br>Macula	Japan	Not specified	Cross-sectional
3	Akashi	2013	84<br>(53)	Myopic	VF+Disc appearance	Cirrus<br>RTVue<br>3DTopcon	RNFL+<br>Macula	Japan	Not specified	Cross-sectional
4	Akashi	2015	52<br>(54)	Myopic	VF+Disc appearance	Cirrus<br>RTVue<br>3DTopcon	RNFL+Macula	Japan	Not specified	Cross-sectional
5	Aptel	2010	40<br>40<br>(40)	Perimetric<br>Suspect	VF+Disc appearance	Cirrus	RNFL	France	Not specified	Cross-sectional<br>Prospective
6	Arifoglu	2015	18<br>(55)	Perimetric	VF+Disc appearance+IOP	RTVue	RNFL+Macula	Turkey	Not specified	Cross-sectional<br>Prospective
7	Arintawati	2013	32<br>81<br>80<br>(68)	Preperimetric<br>Mild<br>Mod to Adv	VF+Disc appearance	RTVue	RNFL+Macula	Japan	38	Cohort<br>Retrospective
8	Asaoka	2017	114<br>(82)	Perimetric	VF+Disc appearance	3DTopcon	RNFL+Macula	Japan	Not specified	Cross-sectional<br>Retrospective
9	Badala	2007	46<br>(46)	Mild	VF	Stratus	RNFL	USA	37	Case-control<br>Retrospective
10	Baskaran	2012	184<br>108<br>58<br>(508)	Perimetric<br>Mild<br>Mod to Adv	VF+Disc appearance	Cirrus	RNFL	Singapore	1	Case-control<br>Consecutive/<br>Prospective
11	Begum	2014	62<br>21<br>(53)	Perimetric<br>Preperimetric	VF+Disc appearance	Cirrus	RNFL+Macula	India	Not specified	Cross-sectional<br>Retrospective
12	Bertuzzi	2014	70<br>(70)	Perimetric	VF+IOP	RTVue	RNFL+Macula	Italy	13	Cross-sectional
13	Bizios	2011	135<br>(125)	Perimetric	Disc appearance	Stratus	RNFL	Sweden	14	Case-control<br>Prospective
14	Blumberg	2016	118<br>52<br>(103)	Perimetric<br>Mild	VF+Disc appearance	Cirrus	RNFL+Macula	USA	16	Case-control<br>Prospective

15	Blumberg	2016	31 (15)	Perimetric	VF+Disc appearance	3DTopcon	RNFL	USA	Not specified	Cohort Prospective
16	Bourne	2004	28 (36)	Perimetric	VF	Stratus	RNFL	USA	8	Cross-sectional Consecutive/ Prospective
17	Brusini	2006	95 (62)	Perimetric	VF+IOP	Stratus	RNFL	Italy	Not specified	Case-control Retrospective
18	Burgansky-Eliash	2005	47 (42)	Perimetric	VF	Stratus	RNFL+Macula	USA	Not specified	Cross-sectional
19	Calvo	2014	156 (182)	Perimetric	VF+IOP	Cirrus	RNFL	Italy	Not specified	Cross-sectional Prospective
20	Cho	2011	49 (43)	Perimetric	VF+Disc appearance	Stratus	RNFL	Korea	6	Case-control Consecutive/ Prospective
21	Choi	2016	71 (86)	Perimetric	VF+Disc appearance	Cirrus	RNFL+Macula	Korea	Not specified	Case-control Retrospective
22	Choi	2013	54 49 (56,22)	Perimetric Myopic	VF+Disc appearance	Cirrus	RNFL+Macula	Korea	6	Case-control Consecutive/ Prospective
23	Dave	2015	76 (80)	Perimetric	VF+Disc appearance	Spectralis	RNFL	India	7	Cross-sectional Retrospective
24	DeLeon-Ortega	2006	79 (149)	Perimetric	VF+Disc appearance	Stratus	RNFL+Macula	USA	25	Case-control Retrospective
25	Garudadri	2012	125 (95)	Perimetric	VF+Disc appearance	Stratus	RNFL	India	20	Case-control Prospective
26	Gunvant	2007	69 (83)	Perimetric	VF	Stratus	RNFL	India	Not specified	Case-control Prospective
27	Gunvant	2009	63 (73)	Perimetric	VF	Stratus	RNFL	India	Not specified	Case-control Prospectively
28	Gyatsho	2008	23 (48)	Perimetric	VF+Disc appearance+IOP	Stratus	RNFL	India	Not specified	Cross-sectional Prospective
29	Hirasawa	2015	89 (89)	Perimetric	VF+Disc appearance	3DTopcon	RNFL	Japan	Not specified	Case-control
30	Hirashima	2013	26 (20)	Preperimetric	VF+Disc appearance	RTVue	RNFL+Macula	Japan	17	Cohort Consecutive/ Prospective
31	Hoesl	2013	33 (32)	Perimetric	VF+Disc appearance+IOP	Spectralis	RNFL	Germany	Not specified	Cross-sectional Retrospective
32	Hollo	2014	66 (50)	Perimetric	VF+Disc appearance+IOP	RTVue	RNFL+Macula	Hungary	10	Cross-sectional Retrospective
33	Hong	2010	49 (59)	Perimetric	VF+Disc appearance+IOP	Cirrus Stratus	RNFL	Korea	Not specified	Cross-sectional

34	Horn	2011	77<br>52<br>50<br>(97)	Preperimetric<br>Mild<br>Mod to Adv	VF+Disc appearance	Spectralis	RNFL	Germany	Not specified	Cross-sectional<br>Retrospective
35	Huang	2011	30<br>47<br>(62)	Preperimetric<br>Mild	VF+Disc appearance+IOP	Cirrus<br>Stratus	RNFL	China	7	Cross-sectional<br>Consecutive/<br>Prospective
36	Huang	2011	146<br>(74)	Perimetric	VF	RTVue	RNFL+Macula	USA	15	Case-control<br>Prospective
37	Hung	2016	41<br>(31)	Myopic	VF+Disc appearance	Stratus<br>Spectralis	Macula	Taiwan	23	Case-control<br>Retrospective
38	Hung	2015	31<br>(41)	Myopic	VF+Disc appearance	Stratus	Macula	Taiwan	Not specified	Case-control<br>Retrospective
39	Hwang	2015	265<br>48<br>110<br>107<br>(181)	Perimetric<br>Preperimetric<br>Mild<br>Mod to Adv	VF+Disc appearance	Cirrus	Macula	Korea	Not specified	Cross-sectional
40	Hwang	2012	80<br>40<br>(80)	Perimetric<br>Mild	VF+Disc appearance	Cirrus	RNFL	Korea	28	Cross-sectional<br>Consecutive/<br>Prospective
41	Jeoung	2010	55<br>(55)	Preperimetric	VF+Disc appearance	Cirrus<br>Stratus	RNFL	Korea	5	Case-control<br>Consecutive/<br>Prospective
42	Jeoung	2014	35<br>(72)	Preperimetric	VF+Disc appearance	Stratus<br>Spectralis	RNFL	Korea	11	Case-control<br>Consecutive/<br>Prospective
43	Jeoung	2013	164<br>142<br>(119)	Mild<br>Mod to Adv	VF+Disc appearance	Cirrus	RNFL+Macula	Korea	1	Cross-sectional
44	Jung	2014	80<br>(100)	Perimetric	VF+Disc appearance	Cirrus	RNFL+Macula	Korea	12	Cross-sectional<br>Prospective
45	Kanamori	2013	40<br>64<br>(75)	Preperimetric<br>Mild	VF+Disc appearance	3DTopcon	RNFL+Macula	Japan	Not specified	Cross-sectional
46	Kang	2012	54<br>(54)	Perimetric	VF	Cirrus	RNFL	Korea	11	Case-control<br>Consecutive/<br>Prospective
47	Kaushik	2011	23<br>32<br>(68)	Perimetric<br>Suspect	VF+Disc appearance	Cirrus<br>Stratus	RNFL	India	Not specified	Cross-sectional<br>Prospective

48	Kiddee	2013	48<br>48<br>(35)	Perimetric<br>Suspect	VF+Disc appearance+IOP	Cirrus	RNFL+Macula	Thailand	7	Cross-sectional<br>Prospective
49	Kim	2014	68<br>(64)	Preperimetric	VF+Disc appearance	3DTopcon	RNFL+Macula	Korea	20	Cross-sectional<br>Retrospective
50	Kim	2011	56<br>21<br>(49,24)	Perimetric<br>Myopic	VF+Disc appearance	RTVue	RNFL+Macula	Korea	5	Case-control<br>Prospective
51	Kim	2013	101<br>(101)	Perimetric	Disc appearance	Cirrus<br>Stratus	RNFL	Korea	35	Case-control<br>Retrospective
52	Kim	2013	52<br>(58)	Perimetric	VF+Disc appearance	RTVue	RNFL+Macula	Korea	5	Cross-sectional<br>Consecutive/<br>Prospective
53	Kim	2013	96<br>(89)	Perimetric	VF	Stratus<br>RTVue	RNFL	Korea	5	Case-control<br>Consecutive/<br>Prospective
54	Kim	2013	34<br>14<br>(42)	Perimetric<br>Preperimetric	VF+Disc appearance	Cirrus<br>3DTopcon	RNFL+Macula	Korea	Not specified	Case-control<br>Retrospective
55	Kim	2016	77<br>(59)	Perimetric	VF+Disc appearance	Spectralis	RNFL+Macula	Korea	Not specified	Case-control
56	Kim	2016	93<br>(102)	Perimetric	VF+Disc appearance	Cirrus	RNFL+Macula	Korea	Not specified	Case-control<br>Consecutive/<br>Prospective
57	Kim	2010	46<br>20<br>(66)	Perimetric<br>Preperimetric	VF+Disc appearance	Cirrus<br>Stratus	RNFL	Korea	5	Case-control<br>Consecutive/<br>Prospective
58	Kim	2010	55<br>83<br>(103)	Mild<br>Mod to Adv	VF+Disc appearance	RTVue	RNFL+Macula	Korea	5	Cross-sectional<br>Consecutive/<br>Prospective
59	Kita	2013	99<br>66<br>33<br>(35)	Perimetric<br>Mild<br>Mod to Adv	VF+Disc appearance	RTVue	RNFL+Macula	Japan	17	Cross-sectional
60	Kita	2013	91<br>(42)	Perimetric	VF+Disc appearance	RTVue	Macula	Japan	29	Case-control
61	Koh	2014	60<br>(50)	Perimetric	VF+Disc appearance	Cirrus	RNFL	Korea	5	Case-control<br>Consecutive/<br>Prospective
62	Kotera	2011	30<br>(35)	Preperimetric	VF+Disc appearance	3DTopcon	Macula	Japan	18	Case-control<br>Prospective

63	Kotowski	2012	63<br>49<br>(51)	Perimetric<br>Suspect	VF+Disc appearance+IOP	Cirrus	RNFL+Macula	USA	Not specified	Cross-sectional<br>Retrospective
64	Larrosa	2015	117<br>(123)	Perimetric	VF	Spectralis	RNFL	Spain	Not specified	Case-control<br>Prospective
65	Lee	2016	60<br>(60)	Perimetric	VF+Disc appearance	Spectralis<br>3DTopcon	RNFL+Macula	Korea	7	Case-control<br>Prospective
66	Lee	2016	75<br>(71)	Perimetric	VF+Disc appearance	Spectralis	RNFL	Korea	43	Case-control<br>Retrospective
67	Lee	2010	88<br>(77)	Perimetric	VF+Disc appearance	Cirrus	RNFL	South Korea	12	Case-control<br>Prospective
68	Leite	2011	126<br>(107)	Perimetric	VF	Cirrus<br>Spectralis<br>RTVue	RNFL	USA	Not specified	Case-control<br>Diagnostic
69	Leite	2010	135<br>(79)	Perimetric	VF	Cirrus	RNFL	USA	Not specified	Case-control<br>Retrospective
70	Leung	2005	30<br>(41)	Mild	VF	Stratus	RNFL	Hong Kong (China)	Not specified	Cross-sectional
71	Leung	2005	41<br>(27)	Perimetric	VF	Stratus	RNFL	Hong Kong (China)	3	Case-control<br>Retrospective
72	Leung	2005	39<br>48<br>(46)	Perimetric<br>Suspect	VF+Disc appearance+IOP	Stratus	RNFL+Macula	Hong Kong (China)	4	Case-control<br>Prospective
73	Leung	2010	121<br>(102)	Perimetric	VF	Cirrus<br>Stratus	RNFL	Hong Kong (China)	6	Cross-sectional<br>Consecutive/<br>Prospective
74	Leung	2008	83<br>(97)	Perimetric	VF	Cirrus<br>Stratus	RNFL	Hong Kong (China)	14	Cross-sectional<br>Consecutive/<br>Prospective
75	Leung	2010	79<br>(76)	Perimetric	VF	Spectralis	RNFL	Hong Kong (China)	4	Cross-sectional<br>Prospective
76	Lisboa	2012	48<br>(86)	Preperimetric	VF+Disc appearance	Spectralis	RNFL	USA	Not specified	Cohort<br>Prospective
77	Lisboa	2012	48<br>(86)	Perimetric	VF+Disc appearance	Spectralis	RNFL	USA	Not specified	Cohort<br>Retrospective
78	Liu	2014	9<br>12<br>(13)	Perimetric<br>Suspect	VF+Disc appearance+IOP	RTVue	RNFL	USA	Not specified	Cross-sectional
79	Lu	2008	89<br>(89)	Perimetric	VF+Disc appearance	Stratus	RNFL	USA	Not specified	Cross-sectional<br>Prospective
80	Medeiros	2012	295<br>38<br>(330)	Perimetric<br>Preperimetric	VF+Disc appearance	Cirrus	RNFL	USA	Not specified	Cross-sectional<br>Retrospective

81	Medeoiros	2004	75 (66)	Perimetric	VF	Stratus	RNFL	USA	19	Cross-sectional Prospective
82	Medeoiros	2005	88 (78)	Perimetric	VF	Stratus	RNFL+Macula	USA	21	Case-control Prospective
83	Mendez-Hernandez	2016	66 (52)	Perimetric	VF+Disc appearance	Spectralis	RNFL	Spain	Not specified	Cross-sectional
84	Monsalve	2016	150 (88)	Perimetric	Disc appearance+IOP	Cirrus	RNFL	Spain	Not specified	Case-control Consecutive/ Prospective
85	Moreno-Montanes	2010	86 (130)	Perimetric	VF+IOP	Cirrus Stratus	RNFL	Spain	Not specified	Cross-sectional Retrospective
86	Moreno-Montanes	2008	111 (69)	Perimetric	VF+IOP	Stratus	RNFL	Spain	Not specified	Cross-sectional Prospective
87	Mori	2010	50<br>24<br>26<br>(35)	Perimetric<br>Mild<br>Mod to Adv	VF+Disc appearance	Stratus RTVue	RNFL+Macula	Japan	11	Cross-sectional
88	Na	2012	38 (61)	Perimetric	VF+Disc appearance	Cirrus	Macula	South Korea	38	Cross-sectional Retrospective
89	Na	2013	105 (68)	Preperimetric	VF+Disc appearance	RTVue	RNFL+Macula	Korea	7	Cohort Consecutive/ Prospective
90	Na	2011	424 (297)	Perimetric	VF	Cirrus	RNFL+Macula	South Korea	17	Case-control Consecutive/ Prospective
91	Na	2013	42 (42)	Perimetric	VF+IOP	Cirrus	RNFL	South Korea	17	Case-control Consecutive/ Prospective
92	Nakatani	2014	64 (40)	Mild	VF+Disc appearance	3DTopcon	RNFL+Macula	Japan	Not specified	Case-control
93	Nomoto	2009	24 (40)	Suspect	VF+Disc appearance	Stratus	RNFL	Japan	Not specified	Cross-sectional
94	Nour-Mahdavi	2013	59 (91)	Perimetric	VF	Cirrus	RNFL+Macula	USA	22	Case-control Prospective
95	Oli	2015	33 (30)	Perimetric	VF+Disc appearance+IOP	Cirrus	Macula	India	15	Case-control Prospective
96	Pablo	2009	53 (128)	Perimetric	Disc appearance+IOP	Stratus	RNFL	Argentina	23	Cross-sectional Prospective
97	Parikh	2010	56 (75)	Mild	VF+Disc appearance	Stratus	Macula	India	19	Cross-sectional Consecutive/ Prospective

98	Parikh	2007	72<br>(96)	Mild	VF+Disc appearance	Stratus	RNFL	India	19	Case-control<br>Consecutive/<br>Prospective
99	Park	2009	100<br>52<br>48<br>(74)	Perimetric<br>Mild<br>Mod to Adv	VF+Disc appearance	Cirrus<br>Stratus	RNFL	South Korea	6	Cross-sectional<br>Consecutive/<br>Prospective
100	Park	2013	68<br>24<br>26<br>(65)	Perimetric<br>Mild<br>Mod to Adv	VF+Disc appearance+IOP	Spectralis	RNFL	Korea	20	Case-control
101	Park	2013	146<br>88<br>(88)	Perimetric<br>Preperimetric	VF+Disc appearance	Cirrus	RNFL	South Korea	21	Cross-sectional<br>Consecutive/<br>Prospective
102	Park	2015	50<br>106<br>(130)	Preperimetric<br>Mild	VF+Disc appearance	Cirrus	RNFL+Macula	Korea	16	Cross-sectional<br>Retrospective
103	Pueyo	2007	73<br>(66)	Perimetric	VF+Disc appearance+IOP	Stratus	RNFL	Spain	Not specified	Cross-sectional
104	Rao	2013	34<br>(60)	Preperimetric	VF+Disc appearance	RTVue	RNFL+Macula	India	36	Cross-sectional
105	Rao	2010	140<br>(74)	Perimetric	VF	RTVue	RNFL+Macula	USA	Not specified	Case-control<br>Retrospective
106	Rao	2012	65<br>(119)	Perimetric	VF+Disc appearance	RTVue	RNFL+Macula	India	Not specified	Cross-sectional
107	Rao	2014	35<br>(94)	Perimetric	VF+Disc appearance	RTVue	RNFL	India	26	Cross-sectional<br>Consecutive/<br>Prospective
108	Rao	2015	179<br>(101)	Perimetric	Disc appearance	RTVue	RNFL+Macula	India	Not specified	Cross-sectional
109	Rao	2012	91<br>(125)	Mild	VF+Disc appearance	RTVue	RNFL+Macula	India	10	Case-control<br>Prospective
110	Rao	2014	106<br>(109)	Perimetric	VF+Disc appearance	RTVue	RNFL	India	26	Cross-sectional<br>Consecutive/<br>Prospective
111	Raza	2014	156<br>(52)	Perimetric	VF	3DTopcon	RNFL+Macula	USA	Not specified	Case-control
112	Rimayanti	2014	41<br>77<br>(45)	Mild<br>Mod to Adv	VF+Disc appearance	RTVue	RNFL+Macula	Japan	46	Cross-sectional<br>Retrospective
113	Rolle	2016	90<br>(23)	Perimetric	VF+Disc appearance	Spectralis	Macula	Italy	13	Cohort<br>Prospective

114	Rolle	2011	116 (52)	Preperimetric	VF+Disc appearance+IOP	RTVue	RNFL+Macula	Italy	11	Cohort Consecutive/ Prospective
115	Schrems	2010	95 89 (57)	Perimetric Preperimetric	VF+Disc appearance+IOP	Stratus	RNFL	Germany	Not specified	Case-control Retrospective
116	Schulze	2015	93 (60)	Perimetric	VF+Disc appearance+IOP	RTVue	RNFL+Macula	Germany	Not specified	Cross-sectional
117	Schulze	2011	93 (60)	Perimetric	VF+Disc appearance+IOP	RTVue	RNFL+Macula	Germany	Not specified	Cross-sectional
118	Sehi	2009	50 (50)	Perimetric	VF+Disc appearance	Stratus RTVue	RNFL	USA	Not specified	Cohort Prospective
119	Sevim	2013	148 (60)	Perimetric	VF+Disc appearance	RTVue	RNFL+Macula	Turkey	46	Cross-sectional
120	Shah	2016	97 100 (94)	Perimetric Suspect	VF+Disc appearance+IOP	Cirrus	RNFL	USA	5	Cross-sectional
121	Shieh	2016	105 31 (58)	Perimetric Mild	VF+Disc appearance	Spectralis	RNFL	USA	59	Cross-sectional Retrospective
122	Shin	2011	26 (24)	Perimetric	VF+Disc appearance+IOP	Stratus Spectralis	RNFL	Korea	5	Cross-sectional Consecutive/ Prospective
123	Shin	2010	76 (36)	Perimetric	VF+Disc appearance	Stratus RTVue	RNFL	Korea	12	Case-control Consecutive/ Prospective
124	Shin	2012	30 (32)	Perimetric	VF+Disc appearance	Cirrus	RNFL	Korea	4	Case-control Retrospective
125	Shin	2014	84 (43)	Mild	VF+Disc appearance	Cirrus	RNFL+Macula	Korea	8	Cross-sectional Retrospective
126	Shin	2015	160 (160)	Perimetric	VF+Disc appearance	Cirrus	RNFL	Korea	4	Case-control Retrospective
127	Shoji	2012	52 (38)	Myopic	VF+Disc appearance	RTVue	RNFL+Macula	Japan	28	Case-control
128	Silva	2013	62 (48)	Perimetric	VF+Disc appearance+IOP	Cirrus	RNFL	Brazil	27	Cross-sectional Prospective
129	Simavli	2015	89 33 (67)	Perimetric Mild	VF+Disc appearance	Spectralis	RNFL	USA	55	Cross-sectional
130	Suh	2013	78 (80)	Mild	VF	Cirrus	RNFL	Korea	15	Case-control Consecutive/ Prospective

131	Sullivan-Mee	2013	50 (50)	Mild	VF	Spectralis	RNFL+Macula	USA	Not specified	Cohort Prospective
132	Sung	2014	40 (20)	Mod to Adv	VF+Disc appearance	3DTopcon	RNFL+Macula	Korea	6	Cross-sectional
133	Sung	2014	37<br>70<br>(72)	Preperimetric<br>Mild	VF+Disc appearance	Cirrus	RNFL+Macula	Korea	5	Cross-sectional Retrospective
134	Sung	2015	220 (242)	Mod to Adv	VF	Cirrus	RNFL+Macula	Korea	Not specified	Case-control
135	Sung	2012	144<br>85<br>(109)	Mild<br>Mod to Adv	VF+Disc appearance	Cirrus	RNFL	Korea	Not specified	Cross-sectional Consecutive/ Prospective
136	Takayama	2012	58<br>38<br>20<br>(47)	Perimetric<br>Mild<br>Mod to Adv	VF+Disc appearance	Cirrus	RNFL+Macula	Japan	42	Case-control
137	Tan	2009	78<br>52<br>(65)	Perimetric<br>Preperimetric	VF+Disc appearance	Stratus<br>RTVue	RNFL+Macula	USA	0	Cross-sectional Prospective
138	Toshev	2017	55 (42)	Mild	VF+Disc appearance	Spectralis	RNFL	Germany	4	Case-control Prospective
139	Ulas	2015	40 (40)	Perimetric	VF+Disc appearance+IOP	Spectralis	RNFL	Turkey	14	Case-control
140	Vessani	2009	61 (57)	Perimetric	VF	Stratus	RNFL+Macula	Brazil	Not specified	Case-control Consecutive/ Prospective
141	Wang	2015	139 (184)	Perimetric	VF+Disc appearance	Stratus	RNFL+Macula	USA	Not specified	Case-control Retrospective
142	Wang	2011	72 (62)	Perimetric	VF+Disc appearance+IOP	RTVue	RNFL	China	8	Case-control Retrospective
143	Wollstein	2005	37 (37)	Perimetric	VF	Stratus	RNFL+Macula	USA	Not specified	Case-control Retrospective
144	Wu	2012	61 (85)	Perimetric	VF+Disc appearance	Spectralis	RNFL	USA	6	Cross-sectional
145	Yang	2015	106<br>68<br>(41)	Perimetric<br>Mild	VF+Disc appearance	Cirrus	RNFL+Macula	China	Not specified	Case-control Retrospective
146	Yoon	2014	96 (87)	Perimetric	VF	Cirrus	RNFL+Macula	Korea	6	Cross-sectional
147	Yuan	2010	34 (42)	Mild	VF+Disc appearance	RTVue	RNFL+Macula	China	8	Cross-sectional Consecutive /Prospective

148	Zhang	2016	28 (28)	Myopic	Disc appearance+IOP	Cirrus RTVue	RNFL+Macula	China	Not specified	Case-control
149	Zhong	2009	38 (40)	Preperimetric	VF+Disc appearance	Stratus	RNFL	China	32	Case-control Prospective
150	Zhong	2010	80 (80)	Perimetric	VF+Disc appearance+IOP	Stratus	RNFL	China	18	Case-control Consecutive/ Prospective
